# Examining dynamic developmental trends: the interrelationship between age-friendly environments and healthy aging in the Chinese population—evidence from China Health and Retirement Longitudinal Study, 2011–2018

**DOI:** 10.1186/s12877-024-05053-7

**Published:** 2024-05-15

**Authors:** Yan Cheng, Zhi-liang Chen, Yue Wei, Ning Gu, Shao-liang Tang

**Affiliations:** 1 Nanjing Hospital of Chinese Medicine, Affiliated to Nanjing University of Chinese Medicine, Nanjing, 210000 People’s Republic of China; 2 Nanjing University of Chinese Medicine, Nanjing, 210023 People’s Republic of China

**Keywords:** Healthy aging, Age-friendly environments, Regional disparities, Within-Person effects, Between-Person effects

## Abstract

**Background:**

The objective of this research is to investigate the dynamic developmental trends between Age-Friendly Environments (AFE) and healthy aging in the Chinese population.

**Methods:**

This study focused on a sample of 11,770 participants from the CHARLS and utilized the ATHLOS Healthy Aging Index to assess the level of healthy aging among the Chinese population. Linear mixed model (LMM) was used to explore the relationship between AFE and healthy aging. Furthermore, a cross-lagged panel model (CLPM) and a random-intercept cross-lagged panel model (RI-CLPM) were used to examine the dynamic developmental trends of healthy aging, taking into account both Between-Person effects and Within-Person effects.

**Results:**

The results from LMM showed a positive correlation between AFE and healthy aging (β = 0.087, *p* < 0.001). There was a positive interaction between the geographic distribution and AFE (central region * AFE: β = 0.031, *p* = 0.038; eastern region * AFE: β = 0.048, *p* = 0.003). In CLPM and RI-CLPM, the positive effect of healthy aging on AFE is a type of Between-Person effects (β ranges from 0.147 to 0.159, *p* < 0.001), while the positive effect of AFE on healthy aging is Within-Person effects (β ranges from 0.021 to 0.024, *p* = 0.004).

**Conclusion:**

Firstly, individuals with high levels of healthy aging are more inclined to actively participate in the development of appropriate AFE compared to those with low levels of healthy aging. Furthermore, by encouraging and guiding individuals to engage in activities that contribute to building appropriate AFE, can elevate their AFE levels beyond the previous average level, thereby improving their future healthy aging levels. Lastly, addressing vulnerable groups by reducing disparities and meeting their health needs effectively is crucial for fostering healthy aging in these populations.

**Supplementary Information:**

The online version contains supplementary material available at 10.1186/s12877-024-05053-7.

## Introduction

To tackle the challenges posed by the rapidly aging population, the World Health Organization (WHO) has introduced the concept of healthy aging. Healthy aging is defined as the process of developing and maintaining the functional ability that enables well-being in older age [1]. It emphasizes the crucial role of a harmonious relationship between individuals and their environment in achieving healthy aging. The environment comprises all the factors in the extrinsic world that form the context of an individual’s life, such as the built environment, people and their relationships, attitudes and values, health and social policies, the systems that support them, and the services that they implement.

Previous research has shown that factors such as good health, a regular lifestyle, and a higher socioeconomic status (SES) are crucial for healthy aging. Firstly, individual health status is positively associated with healthy aging. Past research has found a positive correlation between the number of remaining teeth [2] and the level of healthy aging, while individuals with complex combinations of diseases [3, 4] have the lower level of healthy aging. Secondly, lifestyle habits are also significant factors influencing healthy aging. Several studies [5, 6] have shown a positive association between moderate alcohol consumption, active physical activity, and healthy aging. Conversely, smoking [6, 7] is closely associated with poorer levels of healthy aging. Thirdly, there is a positive association between socioeconomic status and healthy aging, such as higher economic and educational levels [6, 8]. Additionally, research in China has found that experiencing various adverse childhood experience (ACE) is negatively correlated with the likelihood of achieving healthy aging [9]. Overall, researchers have explored the influencing factors of healthy aging from multiple dimensions.

However, there has been limited focus on the relationship between the environment and healthy aging. This is partly because while the definition of healthy aging acknowledges the potential impact of environmental factors, there is no specific comprehensive measure provided. Nevertheless, the WHO has recognized the importance of the environment in individuals' well-being. They have developed guidelines such as the "Global Age-friendly Cities: A Guide" [10] and accompanying AFE Features Checklist, as well as the "Measuring the age-friendliness of cities: a guide to using core indicators" [11]. The WHO has also introduced the concept of Age-Friendly Environments (AFE), which aims to create and maintain environment that support individuals' capabilities throughout their lives, enabling them to age in a healthy and positive way [12].

Previous studies have mainly explored the important roles of AFE in maintaining health status [13–18], promoting regular lifestyle habits [19, 20], enhancing life satisfaction [21–29], and facilitating social participation [30, 31]. However, there are still several gaps in the existing research. Firstly, there is a lack of studies investigating the relationship between AFE and healthy aging. Secondly, the WHO proposed AFE to examine whether the individual's environment is conducive to health. Therefore, does the correlation between AFE and healthy aging vary in different environments, such as urban or rural areas? Thirdly, WHO believes that healthy aging and the environment have a mutual interaction. So, how does this interaction develop over time?

The primary aim of this research is to investigate the dynamic developmental trends between AFE and healthy aging in the Chinese population, based on the concepts of healthy aging and AFE proposed by the WHO. This was accomplished by utilizing four waves of longitudinal data from a large and representative sample in China. A linear mixed model (LMM) was employed to preliminarily explore the relationship between AFE and healthy aging, while also assessing whether this relationship is influenced by urban–rural or regional disparities. Furthermore, both traditional cross-lagged panel model (CLPM) and random-intercept cross-lagged panel model (RI-CLPM) were utilized to examine the dynamic developmental trends between AFE and healthy aging in the Chinese population. Lastly, the underlying factors contributing to these dynamic trends were analyzed by considering Between-Person effects and Within-Person effects.

## Method

### Data sources and participants

Data were obtained from China Health and Retirement Longitudinal Survey (CHARLS) and Atmospheric Composition Analysis Group (ACAG) at Dalhousie University. CHARLS aims to gather high-quality microdata that represent Chinese individuals and households aged 45 and above. These data are crucial for analyzing the challenges posed by an aging population in China and promoting interdisciplinary research on aging. The survey was conducted in four waves: 2011 (Wave 1), 2013 (Wave 2), 2015 (Wave 3), and 2018 (Wave 4), covering 150 counties and 450 communities across 28 provinces, autonomous regions, and municipalities. By 2018, the survey had reached a total of 19,000 participants from 12,400 households. Additionally, the CHARLS Life History Survey was conducted in 2014, which covered the same areas as the CHARLS survey [32]. The study also includes PM_2.5_ data obtained from the ACAG [33], which used satellite and ground monitoring stations to provide detailed information on PM_2.5_ levels. The research was utilized the combined data from CHARLS W1-W4, the 2014 Life History Survey, the Harmonized CHARLS (Version D), and the ACAG for further empirical analysis.

**Fig. 1:**
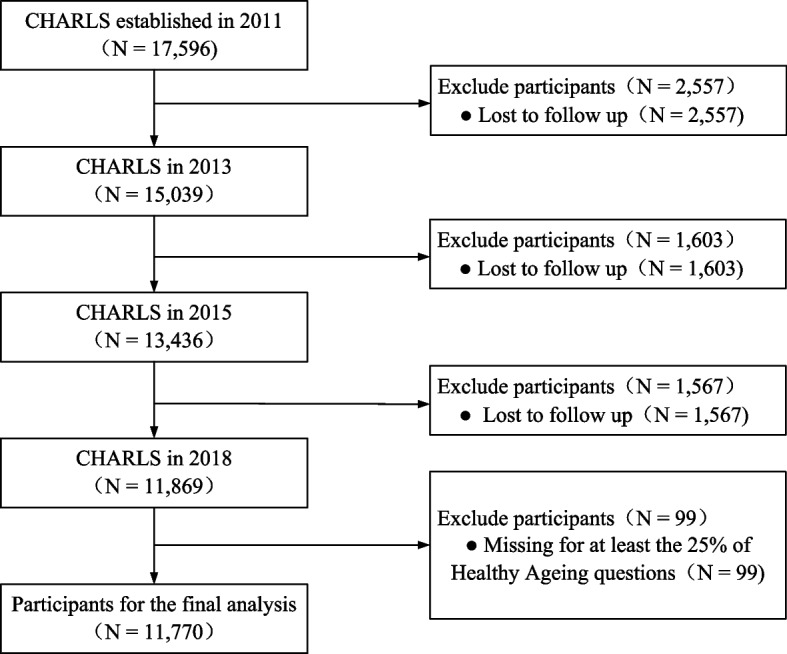
Flowchart of participant selection

A total of 17,596 participants were included in Wave 1 of this study. In the follow-up surveys of Waves 2 to 4, 2,557, 1,603, and 1,567 participants were lost to follow-up or deceased, respectively. Additionally, we excluded 99 participants with missing values greater than 25% for the healthy aging assessment indicators. Finally, a total of 11,770 participants were included in the analysis (Fig. 1).

### Healthy aging (Outcome)

Among the various comprehensive indicators for evaluating healthy aging, the Aging Trajectories of Health: Longitudinal Opportunities and Synergies (ATHLOS) project has developed the most widely used comprehensive indicator for healthy aging. The scale consists of 31 items for CHARLS and is scored using a unidimensional, 2-parameter logistic model (2PLM) of Item Response Theory (IRT) [34], which has been effectively validated [35–37]. In this study, we followed the same method and excluded the Telephone and Walking speed items from the CHARLS survey, as they were not assessed in Wave 1 and Wave 4, respectively. The final scale used in this study was determined based on the aforementioned criteria: Root Mean Square Error of Approximation (RMSEA) < 0.06, Comparative Fit Index (CFI) > 0.95, and Tucker-Lewis Index (TLI) > 0.95, which assessed the adequacy of the measurement scale [34, 38]. Finally, the IRT scores were transformed into T-scores with a mean of 50 and a standard deviation of 10 for further research.

### AFE (Exposure)

Based on the AFE Feature Checklist from the "Global Age-friendly Cities: A Guide" and the "Measuring the Age-Friendliness of Cities: A Guide to Using Core Indicators," as well as previous research findings, we matched 8 indicators from CHARLS to construct the comprehensive evaluation index for AFE (Appendix 1 in the Supplement). All variables are set as binary variables, with a value of 0 for "unfriendly" and a value of 1 for "friendly". Finally, following the method of previous research, we summed up the scores of the 8 indicators to obtain the AFE score, which ranges from 0 to 8. A higher score indicates a more friendly environment [39].

### Covariates

We selected the established factors that have been clearly identified in previous research as control variables for healthy aging (Appendix 2 in the Supplement), including: physical condition (chronic diseases, teeth) [2–4, 34, 35, 38, 40], SES (education, ACE, household income) [3, 4, 6, 8, 9, 34, 35, 40–44], lifestyle habits (smoke, drink) [3, 6, 7, 34, 35, 41, 43, 45], and demographic factors (geographical distribution, urban–rural distribution, age, gender, and marital status) [3, 4, 6, 9, 34, 35, 40, 42].

### Statistical analyses

Continuous variables are described using the mean and standard deviation (SD), while categorical variables are described using frequency and percentage.

We constructed LMM to investigate the relationship between AFE and healthy aging, with participant ID as a random intercept and survey time points as random slopes [46]. Considering the potential influence of covariates on the effect size, three models were considered in this study. Model 1a included only the core variable AFE. Model 1b adjusted for chronic diseases, teeth, household income, education, ACE, smoke, drink, age, gender, marital status, urban–rural distribution, and geographic distribution, based on Model 1a. Model 1c further plus the interaction between AFE and urban–rural distribution, as well as AFE and geographic distribution, based on Model 1b. In order to better explain the interaction effect, we conducted a simple slope analysis. Furthermore, we conducted Variance Inflation Factor (VIF) tests to examine the issue of multicollinearity in the models. The VIF test results showed that the VIF values for all variables in the models were much lower than the critical value of 10, indicating the absence of severe multicollinearity issues (Table S1 in the Supplement).

In our study, we used a traditional CLPM to explore the Between-Person effects [47, 48]. This model includes autoregressive paths, concurrent associations, and bidirectional lagged effects (i.e., the effects from Healthy Aging to AFE and vice versa). To examine the Within-Person effects, we employed the RI-CLPM [47, 48]. Unlike the conventional CLPM, the RI-CLPM distinguishes Within-Person effects and Between-Person effects, allowing us to characterize the Within-Person effects [49].

For CLPM and RI-CLPM, we first estimated unconstrained models where all paths were allowed to vary freely (Model 2a and Model 3a). Then, we imposed constraints on the cross-lagged paths to have the same values across different time points (Model 2b and Model 3b). In the third step, we imposed constraints on the autoregressive paths to have the same values across time (Model 2c and Model 3c). In the fourth step, we imposed constraints on the concurrent paths to have the same values across time (Model 2d and Model 3d). Finally, we imposed constraints on the cross-lagged paths, autoregressive paths, and concurrent paths to have the same values across time (Model 2e and Model 3e). It is important to note that when we impose these equalities across time, the non-standardized coefficients for each path will be identical, but the standardized coefficients will differ. Therefore, in presenting the results, we provided both the non-standardized coefficients and the standardized coefficients.

In order to evaluate the overall fit of the models, we used several indicators including RMSEA, chi-square difference test, CFI, and SRMR (Standardized Root Mean Square Residual). An adequate model fit is indicated when the CFI is greater than or equal to 0.90, RMSEA is less than or equal to 0.08, and SRMR is less than or equal to 0.10. A good model fit is indicated when the CFI is greater than or equal to 0.95, RMSEA is less than or equal to 0.06, and SRMR is less than or equal to 0.08 [50]. We use ΔCFI, ΔRMSEA, and ΔSRMR to compare the differences between the constrained model and the baseline model. When ΔCFI ≤ 0.010 and ΔRMSEA ≤ 0.015 or ΔSRMR ≤ 0.030, we choose the constrained model [51].

Both the CLPM and RI-CLPM models consider covariates. However, incorporating multiple covariates would lead to a more complex model, making it challenging to interpret [52]. Hence, in this study, we included only non-time-varying control variables, including ACE, education, gender, urban–rural distribution, and regional distribution. It is worth noting that in the RI-CLPM model, these control variables are controlled at the random intercept level.

To ensure accurate measurement of healthy aging, we excluded participants who had more than 25% missing values in the comprehensive assessment questionnaire on healthy aging [6]. Assuming that the missingness occurred randomly (Missing at Random, MAR), we employed multiple imputation (MI) to fill in the missing data [3].

We also conducted a series of sensitivity analyses. Firstly, we performed subgroup analyses based on factors such as urban–rural differences, geographical regions, and gender [53]. Secondly, for the assessment of healthy aging, we used a simple summation method instead of IRT [54].

Descriptive statistics and LMM were conducted using R software (Version 4.2.2, R Foundation for Statistical Computing, Vienna, Austria). The mirt package was utilized to score the healthy aging of the Chinese population [55], while the lme4 package was used to build the LMM [56]. The CAR package was used to compute the VIF [57],the mice package was used for MI [58], the interaction package was utilized to plot simple slope graphs [59], and the bruceR package was used to obtain estimates (β), *p*-values, and 95% confidence intervals for fixed and random effects [60]. The CLPM and RI-CLPM models were constructed using Mplus software (Version 8.3, Muthén & Muthén, Los Angeles, USA). A significance level of *p* < 0.05 was used to indicate statistical significance of differences.

## Results

### Baseline characteristics

The IRT model converged successfully with an excellent fit (RMSEA = 0.05, TLI = 0.95, CFI = 0.95 and had a marginal reliability of 0.80). Table 1 summarizes the baseline characteristics of the participants. The study included participants with an average age of 57.57 ± 9.21 years. Among them, males accounted for 46.3% of the total sample, while the urban population represented 34.5% of the participants. In terms of regional distribution, the highest proportion was observed in the West at 32.8%, whereas the lowest proportion was found in the Northeast at 6.8%. On average, the participants had 3.88 ± 1.09 appropriate AFE indicators.

**Table 1:** Baseline descriptive statistics of variables

Variable	Total (*n* = 11,770)
**Number of AFEs, mean (SD)**	3.88 (1.09)
**Number of chronic diseases, mean (SD)**	1.34 (1.37)
**Teeth,** ***N*** (%)	
Tooth loss	869 (7.4)
No tooth loss	10,901 (92.6)
**Household income,** ***N*** (%)	
Q1 (lowest)	2,205 (18.7)
Q2	2,172 (18.5)
Q3	2,243 (19.1)
Q4	2,410 (20.5)
Q5 (highest)	2,740 (23.3)
**Education,** ***N*** (%)	
Primary education	10,531 (89.5)
Secondary education	1,104 (9.4)
Higher education	135 (1.1)
** Number of ACEs, mean (SD)**	2.64 (1.55)
**Smoke,** ***N*** (%)	
None	7,259 (61.7)
Former or current	4,511 (38.3)
**Drink,** ***N*** (%)	
None	7,889 (67.0)
Drink	3,881 (33.0)
** Age, mean (SD)**	57.57 (9.21)
**Gender,** ***N*** (%)	
Male	5,451 (46.3)
Female	6,319 (53.7)
**Marital status,** ***N*** (%)	
Unmarried	1,210 (10.3)
Married	10,560 (89.7)
**Urban–rural distribution,** ***N*** (%)	
Urban	4,056 (34.5)
Rural	7,714 (65.5)
**Regional distribution,** ***N*** (%)	
West	3,860 (32.8)
Northeast	800 (6.8)
Central	3,393 (28.8)
East	3,717 (31.6)

*SD* standard deviation, *AFE* Age-Friendly Environments, *ACEs* adverse childhood experience

### The associations between AFE and healthy aging

**Fig. 2:**
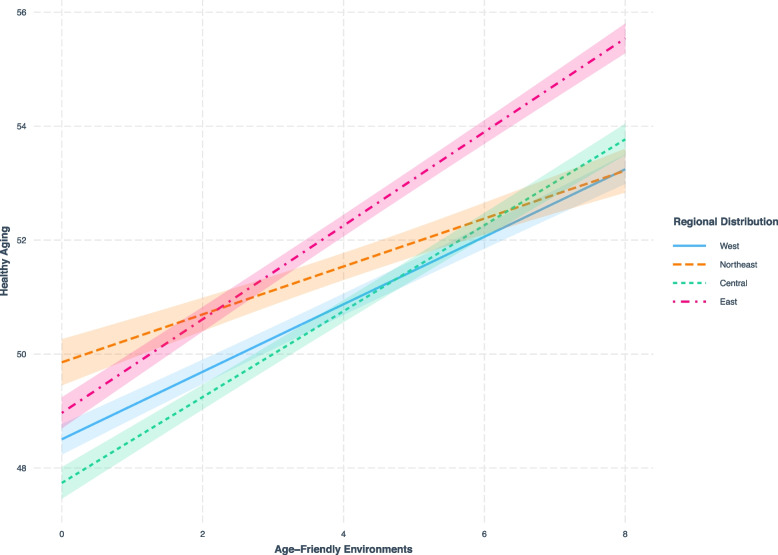
Simple slope analysis

Table 2 provides an initial assessment of the relationship between AFE and healthy aging. Consistent positive associations between AFE and healthy aging were observed across different models (Models 1a-c). According to the multivariable adjusted model (Model 1b), a higher number of appropriate AFE indicators was associated with a higher level of healthy aging (β = 0.087, *p* < 0.001). We also investigated the interaction between urban–rural distribution, geographic distribution, and AFE (Model 1c). No significant interaction was found between urban–rural distribution and AFE (β = 0.014, *p* = 0.318), while an interaction was observed between the geographic distribution and AFE (central region * AFE: β = 0.031, *p* = 0.038; eastern region * AFE: β = 0.048, *p* = 0.003). To further explain the interaction between regional distribution and AFE, we conducted a simple slope test (Fig. 2). The results indicate that in the central region (green dashed line) and the eastern region (red dashed line), as the level of AFE increases, the corresponding level of healthy aging also increases, with a greater upward trend observed in the eastern region.

**Table 2:** Association between AFE and healthy aging of LMM

	**Model 1a**		**Model 1b**		**Model 1c**	
	**β (95% CI)**	***p***	**β (95% CI)**	***p***	**β (95% CI)**	***p***
**Fixed Effects**						
**Core variable**						
**Number of AFEs**	**0.108 (0.100, 0.115)**	< 0.001 ***	**0.087 (0.079, 0.094)**	< 0.001 ***	**0.070 (0.054, 0.086)**	< 0.001 ***
AFE * Urban					0.014 (-0.013, 0.041)	0.318
AFE * Northeast					-0.019 (-0.045, 0.007)	0.160
AFE * Central					**0.031 (0.002, 0.060)**	0.038 *
AFE * East					**0.048 (0.017, 0.079)**	0.003 **
**Covariates**						
**Number of chronic diseases**			**-0.234 (-0.244, -0.224)**	< 0.001 ***	**-0.234 (-0.244, -0.224)**	< 0.001 ***
**Teeth**						
Tooth loss	Reference					
No tooth loss			**0.038 (0.029, 0.048)**	< 0.001 ***	**0.039 (0.029, 0.048)**	< 0.001 ***
**Household income**						
Q1 (lowest)	Reference					
Q2			0.001 (-0.006, 0.009)	0.733	0.001 (-0.006, 0.009)	0.725
Q3			**0.014 (0.006, 0.022)**	< 0.001 ***	**0.014 (0.006, 0.022)**	< 0.001 ***
Q4			**0.023 (0.015, 0.031)**	< 0.001 ***	**0.023 (0.015, 0.031)**	< 0.001 ***
Q5 (highest)			**0.054 (0.044, 0.063)**	< 0.001 ***	**0.054 (0.044, 0.063)**	< 0.001 ***
**Education**						
Primary education	Reference					
Secondary education			**0.072 (0.060, 0.084)**	< 0.001 ***	**0.072 (0.060, 0.084)**	< 0.001 ***
Higher education			**0.047 (0.036, 0.059)**	< 0.001 ***	**0.048 (0.036, 0.059)**	< 0.001 ***
**ACE**						
Number of ACEs			**-0.114 (-0.126, -0.102)**	< 0.001 ***	**-0.114 (-0.126, -0.102)**	< 0.001 ***
**Smoke**						
None	Reference					
Former or current			**-0.033 (-0.047, -0.019)**	< 0.001 ***	**-0.033 (-0.047, -0.019)**	< 0.001 ***
**Drink**						
None	Reference					
Drink			**0.009 (0.001, 0.018)**	0.035 *	**0.009 (0.001, 0.018)**	0.035 *
**Age**						
Age			**-0.211 (-0.224, -0.198)**	< 0.001 ***	**-0.211 (-0.224, -0.198)**	< 0.001 ***
**Gender**						
Male	Reference					
Female			**-0.213 (-0.229, -0.197)**	< 0.001 ***	**-0.213 (-0.229, -0.197)**	< 0.001 ***
**Marital status**						
Unmarried	Reference					
Married			**0.021 (0.011, 0.031)**	< 0.001 ***	**0.021 (0.011, 0.031)**	< 0.001 ***
**Urban–rural distribution**						
Urban	Reference					
Rural			**-0.085 (-0.097, -0.073)**	< 0.001 ***	**-0.098 (-0.126, -0.070)**	< 0.001 ***
**Regional distribution**						
West	Reference					
Northeast			**0.016 (0.004, 0.028)**	0.011 *	**0.034 (0.006, 0.062)**	0.018 *
Central			-0.005 (-0.018, 0.009)	0.502	**-0.035 (-0.066, -0.003)**	0.029 *
East			**0.067 (0.053, 0.081)**	< 0.001 ***	0.022 (-0.011, 0.054)	0.194
**Random Effects**						
	Variance (SD)	Correlation	Variance (SD)	Correlation	Variance (SD)	Correlation
**ID (Intercept)**	59.04 (7.68)		35.86 (5.99)		35.86 (5.99)	
**Wave (Slope)**	0.35 (0.59)	-0.16	0.31 (0.56)	-0.33	0.31 (0.56)	-0.33
**Model fits**						
**R**^**2**^	**Marginal**	**Conditional**	**Marginal**	**Conditional**	**Marginal**	**Conditional**
	0.01	0.64	0.27	0.63	0.27	0.63

*β* standardized coefficient, *CI* confidence interval, *SD* standard deviation, *AFE* Age-Friendly Environments, *ACEs* adverse childhood experience

Marginal: fixed effects; Conditional: fixed and random effects

Model 1a: crude model

Model 1b: adjusted for chronic diseases, teeth, household income, education, ACE, smoke, drink, age, gender, marital status, urban–rural distribution, and geographic distribution

Model 1c: plus the interaction between AFE and urban–rural distribution, as well as AFE and geographic distribution, based on Model 1b

^*^ *p* < .05, ** *p* < .01, *** *p* < .001

### Lagged association between AFE and healthy aging

The fitting results of all CLPM models (Table S2 in the Supplement) are relatively mediocre. The main reason is that the RMSEA values are all above 0.08. For example, in Model 2e, the RMSEA is 0.115. However, according to Orth [48], it is acceptable for some fit indices in CLPM models to be below the threshold. Similarly, in previous studies [61], there are examples of accepting models with RMSEA values exceeding 0.1 for further research. Therefore, we kept the above-mentioned models. After comparing Model 2a-e, Model 2e, which imposes equality constraints on all paths across time, is more favored. Thus, we retained this model for further analysis. The CLPM model results (Table 3) indicate that AFE has a moderate positive predictive effect on healthy aging (β values range from 0.077 to 0.089, *p* < 0.001). Similarly, healthy aging also has a significant positive predictive effect on AFE (β values range from 0.147 to 0.159, *p* < 0.001).

**Table 3:** Cross-Lagged Coefficients of CLPM

CLPM	Effect size	95% CI		*p*
		**Lower**	**Upper**	
**Unstandardized Coefficients**				
AFE → HA	**0.737**	**0.677**	**0.797**	< 0.001 ***
HA → AFE	**0.018**	**0.017**	**0.019**	< 0.001 ***
**Standardized Coefficients**				
AFE1 → HA2	**0.077**	**0.071**	**0.083**	< 0.001 ***
AFE2 → HA3	**0.083**	**0.076**	**0.089**	< 0.001 ***
AFE3 → HA4	**0.089**	**0.081**	**0.096**	< 0.001 ***
HA1 → AFE2	**0.158**	**0.149**	**0.167**	< 0.001 ***
HA2 → AFE3	**0.159**	**0.150**	**0.168**	< 0.001 ***
HA3 → AFE4	**0.147**	**0.138**	**0.155**	< 0.001 ***

*CLPM* cross-lagged panel model, *CI* confidence interval, *AFE1* *AFE2* *AFE3* *AFE4* Age-Friendly Environments at Wave 1, 2, 3, 4, *HA1* *HA2* *HA3* *HA4* healthy aging at Wave 1, 2, 3, 4

Adjusted for ACE, education, gender, urban–rural distribution, and regional distribution

^*^ *p* < .05, ** *p* < .01, *** *p* < .001

The fitting results of all RI-CLPM models (Table S2 in the Supplement) are good. After comparing Model 3a-e, Model 3e, which imposes equality constraints on cross-lagged paths and autoregressive paths across time, is more preferred. Thus, we kept this model for further analysis. The RI-CLPM model results (Table 4) indicate that there is only a significant positive promoting effect of AFE on healthy aging (β values range from 0.021 to 0.024, *p* = 0.004).

**Table 4:** Cross-Lagged Coefficients of RI-CLPM

RI-CLPM	Effect size	95% CI		*p*
		**Lower**	**Upper**	
**Unstandardized Coefficients**				
AFE → HA	**0.144**	**0.062**	**0.227**	0.004 **
HA → AFE	0.000	-0.001	0.002	0.719
**Standardized Coefficients**				
AFE1 → HA2	**0.021**	**0.009**	**0.032**	0.004 **
AFE2 → HA3	**0.023**	**0.010**	**0.036**	0.004 **
AFE3 → HA4	**0.024**	**0.010**	**0.037**	0.004 **
HA1 → AFE2	0.003	-0.011	0.017	0.719
HA2 → AFE3	0.003	-0.010	0.015	0.719
HA3 → AFE4	0.002	-0.008	0.013	0.719

*RI-CLPM* random-intercept cross-lagged panel model, *CI* confidence interval, *AFE* Age-Friendly Environments, *HA* healthy aging

Adjusted for ACE, education, gender, urban–rural distribution, and regional distribution

^*^ *p* < .05, ** *p* < .01, *** *p* < .001

The above results show that the promoting effect of healthy aging on AFE exists in both CLPM and RI-CLPM, while the promoting effect of AFE on healthy aging exists only in RI-CLPM. Consistent with previous research [47, 62], we consider the promoting effect of healthy aging on AFE to be Between-Person effects rather than Within-Person effects, and the promoting effect of AFE on healthy aging to be interpreted as Within-Person effects rather than Between-Person effects.

### Sensitivity analysis

In order to examine the robustness of the findings, a series of sensitivity analyses (Table S3-12 in the Supplement) were conducted. The majority of the results are consistent with the main findings. It is noteworthy that in the subgroups of individuals aged over 65, females, rural residents, individuals from the western, northeastern, and central regions, the results of LMM and CLPM are completely consistent with the main results, but the results of RI-CLPM are no longer significant. For these subgroups, we interpret the effects of AFE on healthy aging and healthy aging on AFE as Between-Person effects.

## Discussion

This study is the first to use microdata from the CHARLS database to assess the dynamic developmental trends between AFE levels and healthy aging in the Chinese population. The results indicate a positive correlation between AFE and healthy aging, with significant positive interactions existing in the central and eastern regions, respectively. Additionally, we found that the promoting effect of healthy aging on AFE is a type of Between-Person effect, while the promoting effect of AFE on healthy aging is a type of Within-Person effect.

The results of the LMM analysis revealed a positive correlation between AFE and healthy aging in the Chinese population. Although there is a lack of research specifically examining the relationship between AFE and healthy aging, previous studies have explored the association between single dimensions of AFE, such as employment and social participation, and healthy aging. These studies consistently found a positive link between employment, social participation, and healthy aging [6, 41, 63]. This may be because work and social engagement contribute to improved physical health, reducing the risk of illness. Moreover, employment and social participation can enhance individuals' social status, promoting psychological well-being and a sense of dignity. Regarding other variables within AFE, although their impact on healthy aging has not been extensively studied, their significance in terms of health should not be disregarded. For instance, PM2.5 pollution may impede the psychological well-being of older adults [64], accelerate cognitive decline in middle-aged and older individuals [65], and increase the risk of premature death. Conversely, a favorable outdoor environment can increase the frequency of social engagement and physical activity, thereby fostering overall well-being [66]. The American Medical Association recognizes the pivotal role of Broadband Internet Access (BIA) in six health domains [67]. Furthermore, the WHO highlights that nearly 2 billion people globally face catastrophic or impoverishing health expenditures, underscoring the fundamental challenge of health inequities in achieving universal health coverage.

In the study of interactions, there is no significant interaction between urban–rural distribution and AFE. This may be due to the continuous improvement of China's urbanization level since the 16th National Congress of the Communist Party of China in 2002, which proposed the concept of "taking the path of urbanization with Chinese characteristics". The urbanization rate has increased from 36.21% in 2000 to 52.57% in 2012, with an average annual growth rate of 1.36 percentage points [68]. By 2018, China's urbanization level reached 59.58% [69]. Additionally, there has been a significant improvement in the living standards of rural residents. The per capita disposable income has risen from 7,394 yuan [70] in 2011 to 14,617 yuan [71] in 2018, and the per capita rural healthcare expenditure has increased from 436.8 yuan [72] to 1,240 yuan [73]. Therefore, although cities have more abundant social and economic resources, and urban populations can better enjoy social security, pensions, healthcare, and other services, the gap between urban and rural areas is narrowing. In terms of the interaction between geographical distribution and AFE, the positive correlation between AFE and healthy aging is stronger in the eastern and central regions than in the western region, with the eastern region showing a stronger correlation than the central region. This may be because, although China has achieved remarkable economic and social development since the reform and opening-up policy, there are still issues of imbalanced and insufficient development. Taking GDP as an example, in 2018, the GDP of the eastern region alone accounted for 53% of the national total [74]. Furthermore, there are significant disparities between the western, northeastern, central regions and the eastern region in terms of healthcare, public resources, and infrastructure [75].

Our results indicate that the promotion of AFE by healthy aging is a Between-Person effect, suggesting that older individuals with higher levels of healthy aging are more likely to experience higher levels of AFE at subsequent time points compared to those with lower levels of healthy aging. This finding can be attributed to several factors. Individuals with higher levels of healthy aging often possess greater intrinsic capabilities, better SES, and healthier lifestyle habits. This enables them to afford expenses related to transportation, healthcare, and retirement, thereby maintaining optimal physical functioning in the long term and exhibiting enhanced learning abilities. Consequently, they have more energy and capacity to actively engage in paid work, social participation, and environmental preservation, thereby fostering an AFE that is conducive to their individual well-being.

Based on our findings, the promotion of healthy aging by AFE is a Within-Person effect, suggesting that encouraging and guiding individuals to engage in building suitable AFE (e.g., paid work, social participation, environmental protection, and fostering a respectful attitude towards the elderly) to achieve AFE levels higher than the previous average (Within-Person effect) can enhance their future level of healthy aging. The reason for this result may be that AFE encompasses multiple key factors from both the physical environment (e.g., accessible public facilities) and the social environment (e.g., active engagement in volunteer activities), providing sufficient support to the Chinese population in multiple aspects. This helps to compensate for or even reverse the gradual loss of intrinsic abilities that occur with age, ultimately achieving a higher level of healthy aging.

The results of the RI-CLPM for subgroups aged over 65, females, rural residents, individuals from the western, northeastern, and central regions did not show significance, indicating that the mutual promotion effect between AFE and healthy aging is a Between-Person effect for these subgroups. Therefore, compared to encouraging and guiding participation in building AFE to achieve levels higher than the previous average level, eliminating age, gender, and regional differences among the population, meeting the diverse health needs of different elderly populations, and steadily improving the health levels of vulnerable groups may be more effective in enhancing the healthy aging levels of the population mentioned above.

However, this study also has several limitations. Firstly, although we combined the "Global Age-friendly Cities: A Guide", the "Measuring the age-friendliness of cities: a guide to using core indicators" and previous research to construct a comprehensive evaluation index for AFE by selecting matching indicators from the CHARLS database, it should be noted that CHARLS is a comprehensive database focusing on the health and elderly care of middle-aged and elderly people in China, rather than a specific survey on AFE. Therefore, some indicators may not fully capture the essence of AFE. Secondly, although the comprehensive evaluation index for healthy aging developed by ATHLOS has been widely validated, it primarily relies on self-assessment and lacks quantitative, objective evaluation indicators, which may introduce recall bias. Thirdly, despite controlling for established factors influencing healthy aging based on existing literature, residual confounding from unmeasured variables cannot be completely ruled out.

## Conclusions

Firstly, individuals with high levels of healthy aging are more inclined to actively participate in the development of appropriate AFE compared to those with low levels of healthy aging. Furthermore, by encouraging and guiding individuals to engage in activities that contribute to building appropriate AFE, such as paid work, social engagement, environmental protection, and fostering a society that respects and values the elderly, can elevate their AFE levels beyond the previous average level (Within-Person effect), thereby improving their future healthy aging levels. Lastly, it is crucial to address vulnerable groups such as the elderly, women, rural residents, and individuals in the western regions by gradually reducing age, gender, urban–rural, and regional disparities, meeting their health needs effectively, enhancing their health status steadily, and fostering healthy aging within these vulnerable populations.

## Statement

All methods in this study on humans described in the manuscript were performed in accordance with national law and the Helsinki Declaration of 1975 and its later amendments.

### Supplementary Information

Supplementary Material 1.

## Data Availability

The datasets used and analyzed during the current study are available in the official website of CHARLS (https://charls.pku.edu.cn/) and ACAG (https://sites.wustl.edu/acag/datasets/surface-pm2-5/).

## Abbreviations

AFE: Age-friendly environments
LMM: Linear mixed model
CLPM: Cross-lagged panel model
RI-CLPM: Random-intercept cross-lagged panel model
WHO: The World Health Organization
SES: Socioeconomic status
CHARLS: China Health and Retirement Longitudinal Survey
ACAG: The Atmospheric Composition Analysis Group
2PLM: 2-Parameter logistic model
IRT: Item Response Theory
RMSEA: Root Mean Square Error of Approximation
SRMR: Standardized Root Mean Square Residual
CFI: Comparative Fit Index
TLI: Tucker-Lewis Index
VIF: Variance Inflation Factor
MI: Multiple imputation
ACE: Adverse childhood experience
