# Supplementary material for: Examining dynamic developmental trends: the interrelationship between age-friendly environments and healthy aging in the Chinese population—evidence from China Health and Retirement Longitudinal Study, 2011–2018
Source: BMC Geriatr. 2024 May 15;24:429. doi: 10.1186/s12877-024-05053-7 (PMC11094897; doi:10.1186/s12877-024-05053-7)
Supplement: Supplementary file 1 — Supplementary Material 1. [file 12877_2024_5053_MOESM1_ESM.docx]

**Section 1 Detailed description of Age-Friendly Environments and covariates**

Appendix 1. Comprehensive evaluation index of Age-Friendly Environments

Age-Friendly Environments (AFE) Features Checklist, as outlined in the "Global Age-friendly Cities: A Guide" published by the World Health Organization (WHO) in 2007, consists of eight domains: Housing, Civic participation and employment, Outdoor spaces and buildings, Community support and health services, Respect and social inclusion, Social participation, Communication and information, and Transportation.

In 2015, the WHO introduced the "Guidelines for Measuring the Degree of Age-friendliness in Cities" to provide a set of core indicators for evaluating AFE. It was noted that although these core indicators may not align perfectly with the 2007 checklist of essential features, they still reflect the key concepts and principles [1]. The core indicators primarily consist of two dimensions: the accessible physical environment and the inclusive social environment. The accessible physical environment includes the neighbourhood walkability, accessibility of public spaces and buildings, accessibility of public transportation vehicles, accessibility of public transportation stops, affordability of housing. The inclusive social environment encompasses positive social attitude toward older people, engagement in volunteer activity, engagement in paid employment, engagement in socio-cultural activity, participation in local decision-making, availability of information, availability of health and social services, economic security.

In previous research [2–16], most of the comprehensive evaluation index for AFE was developed based on the AFE Feature Checklist (only including relevant literature published after 2015, as the core indicators were introduced in that year). This might be because the focus of the 2015 core indicators was on urban environments at the local government level, which limited their usefulness in investigating related issues in rural environments, higher levels of government, or broader geographical areas [1]. Therefore, this study constructed comprehensive evaluation index for AFE based on the 8 dimensions of the 2007 AFE Feature Checklist, combined with the core indicators and previous research, selecting 8 matching indicators from the China Health and Retirement Longitudinal Survey (CHARLS) dataset.

**Housing.** Previous studies [3, 6, 17] have often used the presence of convenient facilities in housing (such as heating, air conditioning, etc.) to examine this indicator. In this study, we adopt the same approach and further set a threshold for housing convenience. Zuluaga's [18] research found that when there are ≥ 2 missing convenient facilities (elevator, hot water, heating, indoor bathroom, bathtub or shower, separate bedroom, automatic washing machine, telephone, and feeling cold frequently), an additional mortality rate associated with the absence of convenient facilities can be observed from the baseline. Therefore, we will use the criterion of ≤ 1 missing convenient facility to measure housing convenience. In the CHARLS, questions I009, I010, I014, I015, I017, I018, and I019 asked about the housing convenience conditions of the participants, including the presence of an elevator, accessibility of pathways, sitting toilet, flushable toilet, availability of running water, bathing facilities, piped coal gas, and natural gas. A value of 1 was assigned for "yes" and 0 for "no". Finally, we summed up the aforementioned variables, with a score range of 0-7. Participants with < 6 convenient conditions in their residences were assigned a value of 0, indicating inconvenience, while those with ≥ 6 conditions were assigned a value of 1, indicating convenience.

**Civic participation and employment.** In previous studies [3–5, 7, 8, 13, 17, 19–25], researchers have measured this indicator by looking at whether people engage in volunteer activities and their employment situation. This article will continue to use the same method. In the CHARLS, participants were asked about their civic participation and employment status, including whether they provide unpaid help to others, participate in volunteer activities, and have a job. We assigned a value of 0 to indicate "no" and a value of 1 to indicate "yes".

**Outdoor spaces and buildings.** Previous studies have used the cleanliness of the outdoor environment [3–7, 13, 17, 23, 26] and air quality [27] as indicators to evaluate this aspect. However, since the CHARLS survey did not include specific questions related to these factors, we had to find an alternative. We obtained PM2.5 data from the ACAG and used it as a measure of outdoor air quality [28]. By using ArcGIS software, we matched the PM2.5 data with the participants at the city level [29]. It is important to note that there was a change in administrative divisions in 2011, where Chaohu City was abolished and its jurisdiction was divided among three other cities. To maintain consistency in our research, we included Chaohu City in our analysis and calculated its PM2.5 data as the average of the three cities it was divided into. Finally, we assigned a value of 1 to an annual average PM2.5 concentration of ≤ 35 μg/m3, in accordance with the "Ambient Air Quality Standards" (GB 3095-2012) [30], and a value of 0 to PM2.5 concentrations > 35 μg/m3.

**Community support and health services.** Previous studies [2, 7, 20] have used the affordability of healthcare services and community support services as a measure to evaluate this aspect. In this study, we will follow the same approach. We measure whether participants face economic barriers in accessing healthcare services by using the concept of Catastrophic Health Expenditure (CHE), which is defined as the ratio of healthcare expenditures to total household expenditures. If this ratio is equal to or greater than 0.4, it indicates that the participants face economic barriers in accessing healthcare services [31], and we assign a value of 0. Otherwise, if the ratio is less than 0.4, we assign a value of 1, indicating no economic barriers. To collect the data, CHARLS asked participants about their household expenditures in the past year in the GE010 questionnaire.

**Respect and social inclusion.** Previous studies [4, 13, 17, 32] have looked at this indicator by considering the willingness of individuals to help each other, and we will adopt the same approach in this study. In the Harmonized CHARLS, information is available on whether participants' relatives (excluding spouses) or friends are willing to provide long-term care in the future (R1FTRHLP). We assign a value of 0 to indicate "not willing" and a value of 1 to indicate "willing".

**Social participation.** Previous studies [3–5, 7, 13, 17, 19–21, 23–25] have often used participation in leisure activities as a way to measure this indicator, and we will adopt the same approach in this study. In the CHARLS survey (S1, S2, S4, S5, S9, and S10 of the DA056 questionnaire), participants were asked about their social engagement in various activities, including socializing with friends, playing mahjong, chess, card games, dancing, exercising, stock trading, and internet use. We assigned a value of 0 to indicate "no participation" and 1 to indicate "participation".

**Communication and information.** Previous studies [27, 33–35] have used the presence of computers and internet access in households as a way to assess this indicator, and this article will adopt the same approach. In the CHARLS (I024), participants were asked whether their homes have broadband internet access. We assigned a value of 0 to those who answered "no" and a value of 1 to those who answered "yes".

**Transportation.** Previous studies [5, 7, 19–23, 25, 26, 36] have used the affordability of public transportation, including taxis and buses, as a measure of this indicator. We will continue to use this method in our study. We measure the affordability of local transportation for participants by calculating the ratio of local transportation expenses to total household expenses. In previous research [37], Chilean scholars used a threshold of < 5% to determine affordability. However, since Chile has had a consistently higher GDP than China (e.g., Chile's per capita GDP was 15,901 in 2018, while China's was only 9,761), we set the threshold for affordability of local transportation in China at 2.5%. In the CHARLS survey, participants were asked about their household expenditures in the past month in module GE009. We consider a ratio ≥ 2.5% as unaffordable local transportation and assign a value of 0, while a ratio < 2.5% is considered affordable and assigned a value of 1.

Appendix 2. Covariates

We selected the established factors that have been clearly identified in previous research as control variables for healthy aging, including: physical condition (chronic diseases, teeth), SES (household income, education, ACE), lifestyle habits (smoke, drink), and demographic factors (age, gender, marital status, urban-rural distribution, and regional distribution).

**Chronic diseases.** In the CHARLS study, the following diseases are defined as chronic diseases (DA007): hypertension, dyslipidemia, diabetes or elevated blood glucose, malignant tumors such as cancer, chronic lung diseases such as chronic bronchitis or emphysema, pulmonary heart disease, liver disease, heart disease, stroke, kidney disease, gastrointestinal or digestive system diseases, emotional and mental health issues, memory-related diseases, arthritis or rheumatism, and asthma. The number of chronic diseases is considered a continuous variable, where a higher value indicates a greater number of chronic diseases.

**Teeth.** In the CHARLS survey, participants were asked about their tooth loss status (DA040). Tooth loss was coded as 0 to indicate that the participants had experienced tooth loss, while a code of 1 was assigned to indicate that their teeth were still intact.

**Household income.** The Harmonized CHARLS provides the total sum of all income at the household level (HHWITOT), which encompasses various sources such as wages, bonuses, capital gains, pension payments, government transfers, income from other household members, and miscellaneous sources. In our analysis, we calculate the per capita income by dividing the household total income by the number of household members. We then adjust the per capita income for inflation using the Consumer Price Index (CPI) with a base year of 2010 [38]. Finally, we assign a value ranging from 1 to 5 based on the household's income percentile after performing quintile classification.

**Education.** The Harmonized CHARLS provides information on participants' educational background (RAEDUCL). We assign a value of 0 to those who received primary education (junior high school or below), a value of 1 to those with secondary education (high school or vocational school), and a value of 2 to those with higher education (college or above).

**ACE.** The Harmonized CHARLS provides information on participants' childhood (under 16 years old) physical health status (RAHLTCOM) and childhood family economic status (RAFINACOM). We assigned a value of 1 to indicate poorer childhood health and economic conditions, and a value of 0 to indicate better childhood health and economic conditions. The 2014 Life History Survey asked about various circumstances during participants' childhood, such as whether their parents were bedridden for a long time (H17), had severe disabilities (H18), had mental disorders (H19), had poor marital relationships (J9), had unhealthy habits (including: H9: alcoholism, smoking, drug use, gambling; H15: difficulty maintaining employment, frequent lying, fighting, involvement in illegal activities, arrest or imprisonment), experienced divorce (A10), or experienced the death of a parent (A2). It also asked whether participants experienced parental abuse during childhood (K1), whether their living environment was unsafe at night (D1), and whether they were bullied by neighborhood children (F1) or classmates (F3). A value of 1 was assigned to indicate "yes" and a value of 0 was assigned to indicate "no" for these circumstances. Finally, we summed up the above variables to obtain a new variable called "Number of ACEs", with a score ranging from 0 to 12. A higher score indicates a higher number of ACEs [39].

**Smoke.** The Harmonized CHARLS provides information on the participants' smoking status (RWSMOKEV). We assign a value of 0 to those who have never smoked and a value of 1 to those who have ever or currently smoke.

**Drink.** The Harmonized CHARLS provides information on the participants' drinking status (RWDRINKL). We assign a value of 0 to those who have never consumed alcohol and a value of 1 to those who have ever or currently consume alcohol.

**Age.** The Harmonized CHARLS provides the age of the participants. To reduce unnecessary missing values, we used the baseline age (R1AGEY) and added 2, 4, and 7 to generate the respective age values for each wave.

**Gender.** The Harmonized CHARLS provides data on the gender of the survey participants (RAGENDER). We assign a value of 0 to represent males, and a value of 1 to represent females.

**Marital status.** The Harmonized CHARLS provides data on the marital status of the survey participants (RWMSTATH). We assign a value of 0 to represent individuals who are unmarried, separated, divorced, or widowed, and a value of 1 to represent those who are married.

**Urban and rural distribution.** The Harmonized CHARLS provides data on the urban-rural distribution of participants at the baseline (H1RURAL) survey. In this dataset, urban areas are coded as 0, while rural areas are coded as 1.

**Regional distribution.** The participants' provinces were determined based on the Community ID in CHARLS, and they were categorized into four major regions: Western, Northeastern, Central, and Eastern [40]. We assigned values of 1 to 4 to represent these regions accordingly.

**Section2 Supplementary Tables**

Table S1. Results of multicollinearity test

| **Variables** | **GVIF** | **DF** | **GVIF^(1/(2*Df))** |
| --- | --- | --- | --- |
| Number of AFEs | 1.10 | 1 | 1.05 |
| Number of chronic diseases | 1.22 | 1 | 1.10 |
| Teeth | 1.12 | 1 | 1.06 |
| Household income | 1.08 | 4 | 1.01 |
| Education | 1.10 | 2 | 1.02 |
| Number of ACEs | 1.05 | 1 | 1.03 |
| Drink | 1.13 | 1 | 1.06 |
| Smoke | 1.80 | 1 | 1.34 |
| Age | 1.55 | 1 | 1.24 |
| Gender | 1.92 | 1 | 1.39 |
| Marital | 1.10 | 1 | 1.05 |
| Urban-rural distribution | 1.08 | 1 | 1.04 |
| Regional distribution | 1.05 | 3 | 1.01 |

Note: GVIF: generalized variance-inflation factors; AFE: Age-Friendly Environments; ACEs: adverse childhood experience.

Table S2.Fitted results of CLPM and RI-CLPM models in the full sample

| **Models** | **Model fits** | | | | | |  |  | **Pairs** |  | | |
| --- | --- | --- | --- | --- | --- | --- | --- | --- | --- | --- | --- | --- |
|  | **χ2** | **df** | ***p*** | **RMSEA** | **95%CI** | | **CFI** | **SRMR** |  | **ΔRMSEA** | **ΔCFI** | **ΔSRMR** |
|  |  |  |  |  | **Lower** | **Upper** |  |  |  |  |  |  |
| **CLPM** | | | | | | | | | | | | |
| Model 2a | 3,250.36 | 12 | < 0.001 *** | 0.151 | 0.147 | 0.156 | 0.905 | 0.051 |  |  |  |  |
| Model 2b | 3,266.90 | 16 | < 0.001 *** | 0.131 | 0.128 | 0.135 | 0.904 | 0.051 | M2b vs. M2a | 0.020 | 0.001 | 0.000 |
| Model 2c | 3,404.81 | 16 | < 0.001 *** | 0.134 | 0.130 | 0.138 | 0.900 | 0.056 | M2c vs. M2a | 0.017 | 0.005 | 0.005 |
| Model 2d | 3,263.55 | 14 | < 0.001 *** | 0.140 | 0.136 | 0.145 | 0.904 | 0.051 | M2d vs. M2a | 0.011 | 0.001 | 0.000 |
| Model 2e | **3,428.19** | **22** | **< 0.001 ***** | **0.115** | **0.111** | **0.118** | **0.900** | **0.057** | M2e vs. M2a | **0.036** | **0.005** | **0.006** |
| **RI-CLPM** | | | | | | | | | | | | |
| Model 3a | 452.74 | 39 | < 0.001 *** | 0.030 | 0.028 | 0.033 | 0.988 | 0.022 |  |  |  |  |
| Model 3b | 476.73 | 43 | < 0.001 *** | 0.029 | 0.027 | 0.032 | 0.987 | 0.022 | M3b vs. M3a | 0.001 | 0.001 | 0.000 |
| Model 3c | 628.33 | 43 | < 0.001 *** | 0.034 | 0.032 | 0.036 | 0.983 | 0.036 | M3c vs. M3a | 0.004 | 0.005 | 0.014 |
| Model 3d | 504.80 | 41 | < 0.001 *** | 0.031 | 0.029 | 0.033 | 0.986 | 0.021 | M3d vs. M3a | 0.001 | 0.002 | 0.001 |
| Model 3e | **668.49** | **49** | **< 0.001 ***** | **0.033** | **0.031** | **0.035** | **0.982** | **0.037** | M3e vs. M3a | **0.003** | **0.006** | **0.015** |

Note: CLPM: cross-lagged panel model; RI-CLPM: random-intercept cross-lagged panel model; SRMR: standardized root mean squared residual; RMSEA: root mean square error of approximation; CFI: comparative fit index; CI: confidence interval.

Model 2a-3a: unconstrained baseline model; Model 2b-3b: constrained cross-lagged paths; Model 2c-3c: constrained autoregressive paths; Model 2d-3d: constrained concurrent paths; Model 2e-3e: constrained all paths.

Table S3. Sensitivity analysis of associations between AFE and Healthy Ageing stratified by age in LMM

| **Age** | **Age ≥ 65** | | **Age < 65** | |
| --- | --- | --- | --- | --- |
|  | **β (95% CI)** | ***p*** | **β (95% CI)** | ***p*** |
| **Fixed Effects** | | | | |
| **AFE** | | | | |
| Number of AFEs | **0.094 (0.079, 0.110)** | **< 0.001 ***** | **0.091 (0.082, 0.099)** | **< 0.001 ***** |
| **Chronic diseases** | | | | |
| Number of chronic diseases | **-0.207 (-0.229, -0.185)** | **< 0.001 ***** | **-0.259 (-0.271, -0.248)** | **< 0.001 ***** |
| **Teeth** | | | | |
| Tooth loss | **Reference** | | | |
| No tooth loss | **0.069 (0.048, 0.090)** | **< 0.001 ***** | **0.038 (0.028, 0.048)** | **< 0.001 ***** |
| **Household income** | | | | |
| Q1 (lowest) | **Reference** | | | |
| Q2 | **0.019 (0.002, 0.037)** | **0.030 *** | -0.007 (-0.016, 0.002) | 0.137 |
| Q3 | 0.013 (-0.004, 0.030) | 0.137 | **0.014 (0.004, 0.023)** | 0.004 ** |
| Q4 | **0.029 (0.012, 0.047)** | **< 0.001 ***** | **0.023 (0.013, 0.032)** | **< 0.001 ***** |
| Q5 (highest) | **0.062 (0.041, 0.082)** | **< 0.001 ***** | **0.053 (0.042, 0.064)** | **< 0.001 ***** |
| **Education** | | | | |
| Primary education | **Reference** | | | |
| Secondary education | **0.060 (0.032, 0.087)** | **< 0.001 ***** | **0.094 (0.081, 0.108)** | **< 0.001 ***** |
| Higher education | **0.048 (0.020, 0.075)** | **< 0.001 ***** | **0.053 (0.040, 0.067)** | **< 0.001 ***** |
| **ACE** | | | | |
| Number of ACEs | **-0.107 (-0.135, -0.080)** | **< 0.001 ***** | **-0.116 (-0.129, -0.102)** | **< 0.001 ***** |
| **Smoke** | | | | |
| None | **Reference** | | | |
| Former or current | -0.023 (-0.052, 0.006) | 0.113 | **-0.040 (-0.057, -0.023)** | **< 0.001 ***** |
| **Drink** | | | | |
| None | **Reference** | | | |
| Former or current | **0.029 (0.011, 0.048)** | **0.002 **** | 0.005 (-0.006, 0.015) | 0.363 |
| **Gender disparities** | | | | |
| Male | **Reference** | | | |
| Female | **-0.204 (-0.238, -0.170)** | **< 0.001 ***** | **-0.209 (-0.228, -0.190)** | **< 0.001 ***** |
| **Marital status** | | | | |
| Unmarried | **Reference** | | | |
| Married | **0.033 (0.011, 0.056)** | **0.004 **** | **0.028 (0.017, 0.039)** | **< 0.001 ***** |
| **Urban-rural distribution** | | | | |
| Urban | **Reference** | | | |
| Rural | **-0.082 (-0.111, -0.054)** | **< 0.001 ***** | **-0.087 (-0.101, -0.073)** | **< 0.001 ***** |
| **Regional distribution** | | | | |
| West | **Reference** | | | |
| Northeast | -0.011 (-0.040, 0.017) | 0.442 | **0.021 (0.006, 0.035)** | **0.005 **** |
| Central | -0.008 (-0.039, 0.023) | 0.625 | -0.006 (-0.022, 0.009) | 0.411 |
| East | **0.056 (0.025, 0.088)** | **< 0.001 ***** | **0.067 (0.051, 0.083)** | **< 0.001 ***** |
| **Random Effects** | | | | |
|  | Variance (SD) | Correlation | Variance (SD) | Correlation |
| **ID (Intercept)** | 45.90 (6.78) |  | 34.36 (5.86) |  |
| **Wave (Slope)** | 0.42 (0.65) | -0.27 | 0.27 (0.52) | -0.34 |
| **Model fits** | | | | |
| **R^2^** | **Marginal** | **Conditional** | **Marginal** | **Conditional** |
|  | 0.18 | 0.62 | 0.22 | 0.60 |

Note: β, standardized coefficient; CI: confidence interval; SD, standard deviation; AFE: Age-Friendly Environments; ACEs: adverse childhood experience.

Marginal: fixed effects; Conditional: fixed and random effects.

Adjusted for chronic diseases, teeth, household income, education, ACE, smoke, drink, gender, marital status, urban-rural distribution, and geographic distribution.

* p < .05, ** p < .01, *** p < .001.

Table S4. Sensitivity analysis of associations between AFE and Healthy Ageing stratified by age in CLPM和RI-CLPM

| **Age** | **Age ≥ 65** | | **Age < 65** | |
| --- | --- | --- | --- | --- |
|  | **Effect size (95% CI)** | ***p*** | **Effect size (95% CI)** | ***p*** |
| **CLPM** | | | | |
| **Unstandardized model results** | | | | |
| AFE→HA | **0.653 (0.516, 0.789)** | **< 0.001 ***** | **0.608 (0.542, 0.675)** | **< 0.001 ***** |
| HA→AFE | **0.014 (0.012, 0.016)** | **< 0.001 ***** | **0.016 (0.015, 0.017 )** | **< 0.001 ***** |
| **Standardized model results** | | | | |
| AFE1→HA2 | **0.064 (0.050, 0.077)** | **< 0.001 ***** | **0.065 (0.058, 0.073)** | **< 0.001 ***** |
| AFE2→HA3 | **0.069 (0.054, 0.083)** | **< 0.001 ***** | **0.070 (0.063, 0.078)** | **< 0.001 ***** |
| AFE3→HA4 | **0.075 (0.059, 0.090)** | **< 0.001 ***** | **0.075 (0.067, 0.084)** | **< 0.001 ***** |
| HA1→AFE2 | **0.135 (0.115, 0.155)** | **< 0.001 ***** | **0.139 (0.128, 0.149)** | **< 0.001 ***** |
| HA2→AFE3 | **0.133 (0.114, 0.153)** | **< 0.001 ***** | **0.140 (0.129, 0.150)** | **< 0.001 ***** |
| HA3→AFE4 | **0.125 (0.107, 0.144)** | **< 0.001 ***** | **0.128 (0.118, 0.137)** | **< 0.001 ***** |
| **RI-CLPM** | | | | |
| **Unstandardized model results** | | | | |
| AFE→HA | 0.220 (0.035, 0.406) | 0.051 | **0.112 (0.020, 0.203)** | **0.044 *** |
| HA→AFE | -0.001 (-0.005, 0.003) | 0.622 | 0.001 (-0.001, 0.003) | 0.432 |
| **Standardized model results** | | | | |
| AFE1→HA2 | 0.030 (0.005, 0.055) | 0.051 | **0.016 (0.003, 0.029)** | **0.044 *** |
| AFE2→HA3 | 0.034 (0.005, 0.063) | 0.051 | **0.018 (0.003, 0.033)** | **0.044 *** |
| AFE3→HA4 | 0.034 (0.005, 0.062) | 0.051 | **0.019 (0.003, 0.034)** | **0.044 *** |
| HA1→AFE2 | -0.009 (-0.039, 0.021) | 0.622 | 0.008 (-0.008, 0.023) | 0.432 |
| HA2→AFE3 | -0.008 (-0.035, 0.019) | 0.622 | 0.007 (-0.007, 0.021) | 0.432 |
| HA3→AFE4 | -0.007 (-0.030, 0.016) | 0.622 | 0.006 (-0.006, 0.018) | 0.432 |

Note: CLPM: cross-lagged panel model; RI-CLPM: random-intercept cross-lagged panel model; CI: confidence interval; AFE1, AFE2, AFE3, AFE4: Age-Friendly Environments at Wave 1, 2, 3, 4; HA1, HA2, HA3, HA4: healthy aging at Wave 1, 2, 3, 4.

Adjusted for ACE, education, gender, urban-rural distribution, and regional distribution.

* p < .05, ** p < .01, *** p < .001.

Table S5. Sensitivity analysis of associations between AFE and Healthy Ageing stratified by sex in LMM

| **Gender disparities** | **Male** | | **Female** | |
| --- | --- | --- | --- | --- |
|  | **β (95% CI)** | ***p*** | **β (95% CI)** | ***p*** |
| **Fixed Effects** | | | | |
| **AFE** | | | | |
| Number of AFEs | **0.095 (0.084, 0.106)** | **< 0.001 ***** | **0.083 (0.073, 0.093)** | **< 0.001 ***** |
| **Chronic diseases** | | | | |
| Number of chronic diseases | **-0.228 (-0.242, -0.213)** | **< 0.001 ***** | **-0.249 (-0.263, -0.236)** | **< 0.001 ***** |
| **Teeth** | | | | |
| Tooth loss | **Reference** | | | |
| No tooth loss | **0.033 (0.019, 0.047)** | **< 0.001 ***** | **0.044 (0.031, 0.057)** | **< 0.001 ***** |
| **Household income** | | | | |
| Q1 (lowest) | **Reference** | | | |
| Q2 | -0.002 (-0.014, 0.010) | 0.712 | 0.005 (-0.006, 0.015) | 0.416 |
| Q3 | **0.017 (0.006, 0.029)** | **0.004 **** | **0.012 (0.001, 0.023)** | **0.028 *** |
| Q4 | **0.031 (0.018, 0.043)** | **< 0.001 ***** | **0.018 (0.006, 0.029)** | **0.002 **** |
| Q5 (highest) | **0.057 (0.042, 0.071)** | **< 0.001 ***** | **0.053 (0.041, 0.066)** | **< 0.001 ***** |
| **Education** | | | | |
| Primary education | **Reference** | | | |
| Secondary education | **0.081 (0.063, 0.099)** | **< 0.001 ***** | **0.067 (0.050, 0.083)** | **< 0.001 ***** |
| Higher education | **0.063 (0.046, 0.081)** | **< 0.001 ***** | **0.031 (0.015, 0.047)** | **< 0.001 ***** |
| **ACE** | | | | |
| Number of ACEs | **-0.115 (-0.133, -0.097)** | **< 0.001 ***** | **-0.116 (-0.132, -0.100)** | **< 0.001 ***** |
| **Smoke** | | | | |
| None | **Reference** | | | |
| Former or current | **-0.028 (-0.042, -0.013)** | **< 0.001 ***** | **-0.024 (-0.038, -0.009)** | **0.001 **** |
| **Drink** | | | | |
| None | **Reference** | | | |
| Former or current | **0.035 (0.022, 0.047)** | **< 0.001 ***** | **-0.018 (-0.028, -0.007)** | **< 0.001 ***** |
| **Age** | | | | |
| Age | **-0.203 (-0.223, -0.183)** | **< 0.001 ***** | **-0.220 (-0.239, -0.202)** | **< 0.001 ***** |
| **Marital status** | | | | |
| Unmarried | **Reference** | | | |
| Married | 0.010 (-0.004, 0.025) | 0.172 | **0.028 (0.014, 0.042)** | **< 0.001 ***** |
| **Urban-rural distribution** | | | | |
| Urban | **Reference** | | | |
| Rural | **-0.075 (-0.093, -0.057)** | **< 0.001 ***** | **-0.098 (-0.114, -0.081)** | **< 0.001 ***** |
| **Regional distribution** | | | | |
| West | **Reference** | | | |
| Northeast | -0.004 (-0.023, 0.015) | 0.656 | **0.034 (0.017, 0.051)** | **< 0.001 ***** |
| Central | -0.008 (-0.028, 0.013) | 0.464 | -0.003 (-0.021, 0.016) | 0.781 |
| East | **0.074 (0.053, 0.095)** | **< 0.001 ***** | **0.062 (0.044, 0.081)** | **< 0.001 ***** |
| **Random Effects** | | | | |
|  | Variance (SD) | Correlation | Variance (SD) | Correlation |
| **ID (Intercept)** | 35.80 (5.98) |  | 35.72 (5.98) |  |
| **Wave (Slope)** | 0.28 (0.53) | -0.26 | 0.33 (0.58) | -0.38 |
| **Model fits** | | | | |
| **R^2^** | **Marginal** | **Conditional** | **Marginal** | **Conditional** |
|  | 0.23 | 0.61 | 0.24 | 0.61 |

Note: β, standardized coefficient; CI: confidence interval; SD, standard deviation; AFE: Age-Friendly Environments; ACEs: adverse childhood experience.

Marginal: fixed effects; Conditional: fixed and random effects.

Adjusted for chronic diseases, teeth, household income, education, ACE, smoke, drink, age, marital status, urban-rural distribution, and geographic distribution.

* p < .05, ** p < .01, *** p < .001.

Table S6. Sensitivity analysis of associations between AFE and Healthy Ageing stratified by sex in CLPM和RI-CLPM

| **Gender disparities** | **Male** | | **Female** | |
| --- | --- | --- | --- | --- |
|  | **Effect size (95% CI)** | ***p*** | **Effect size (95% CI)** | ***p*** |
| **CLPM** | | | | |
| **Unstandardized model results** | | | | |
| AFE→HA | **0.761 (0.671, 0.850)** | **< 0.001 ***** | **0.713 (0.633, 0.794)** | **< 0.001 ***** |
| HA→AFE | **0.018 (0.017, 0.020)** | **< 0.001 ***** | **0.018 (0.016, 0.019)** | **< 0.001 ***** |
| **Standardized model results** | | | | |
| AFE1→HA2 | **0.081 (0.071, 0.091)** | **< 0.001 ***** | **0.076 (0.068, 0.085)** | **< 0.001 ***** |
| AFE2→HA3 | **0.087 (0.077, 0.097)** | **< 0.001 ***** | **0.082 (0.073, 0.092)** | **< 0.001 ***** |
| AFE3→HA4 | **0.091 (0.080, 0.101)** | **< 0.001 ***** | **0.090 (0.079, 0.100)** | **< 0.001 ***** |
| HA1→AFE2 | **0.154 (0.141, 0.167)** | **< 0.001 ***** | **0.156 (0.144, 0.169)** | **< 0.001 ***** |
| HA2→AFE3 | **0.159 (0.146, 0.172)** | **< 0.001 ***** | **0.153 (0.141, 0.166)** | **< 0.001 ***** |
| HA3→AFE4 | **0.145 (0.133, 0.157)** | **< 0.001 ***** | **0.142 (0.130, 0.153)** | **< 0.001 ***** |
| **RI-CLPM** | | | | |
| **Unstandardized model results** | | | | |
| AFE→HA | **0.182 (0.055, 0.308)** | **0.018 *** | 0.119 (0.010, 0.227) | 0.073 |
| HA→AFE | 0.001 (-0.002, 0.004) | 0.479 | 0.000 (-0.003, 0.002) | 0.91 |
| **Standardized model results** | | | | |
| AFE1→HA2 | **0.025 (0.008, 0.043)** | **0.018 *** | 0.017 (0.001, 0.033) | 0.072 |
| AFE2→HA3 | **0.028 (0.009, 0.048)** | **0.018 *** | 0.019 (0.002, 0.037) | 0.073 |
| AFE3→HA4 | **0.028 (0.008, 0.048)** | **0.018 *** | 0.020 (0.002, 0.039) | 0.073 |
| HA1→AFE2 | 0.009 (-0.012, 0.029) | 0.479 | -0.001 (-0.021, 0.018) | 0.91 |
| HA2→AFE3 | 0.008 (-0.011, 0.028) | 0.479 | -0.001 (-0.018, 0.015) | 0.91 |
| HA3→AFE4 | 0.007 (-0.009, 0.023) | 0.479 | -0.001 (-0.015, 0.013) | 0.91 |

Note: CLPM: cross-lagged panel model; RI-CLPM: random-intercept cross-lagged panel model; CI: confidence interval; AFE1, AFE2, AFE3, AFE4: Age-Friendly Environments at Wave 1, 2, 3, 4; HA1, HA2, HA3, HA4: healthy aging at Wave 1, 2, 3, 4.

Adjusted for ACE, education, urban-rural distribution, and regional distribution.

* p < .05, ** p < .01, *** p < .001.

Table S7. Sensitivity analysis of associations between AFE and Healthy Ageing stratified by urban and rural in LMM

| **Urban-rural disparities** | **Urban** | | **Rural** | |
| --- | --- | --- | --- | --- |
|  | **β (95% CI)** | ***p*** | **β (95% CI)** | ***p*** |
| **Fixed Effects** | | | | |
| **AFE** | | | | |
| Number of AFEs | **0.084 (0.071, 0.097)** | **< 0.001 ***** | **0.088 (0.079, 0.097)** | **< 0.001 ***** |
| **Chronic diseases** | | | | |
| Number of chronic diseases | **-0.237 (-0.254, -0.220)** | **< 0.001 ***** | **-0.237 (-0.249, -0.224)** | **< 0.001 ***** |
| **Teeth** | | | | |
| Tooth loss | **Reference** | | | |
| No tooth loss | **0.055 (0.039, 0.070)** | **< 0.001 ***** | **0.030 (0.018, 0.042)** | **< 0.001 ***** |
| **Household income** | | | | |
| Q1 (lowest) | **Reference** | | | |
| Q2 | 0.001 (-0.013, 0.014) | 0.939 | 0.002 (-0.008, 0.011) | 0.721 |
| Q3 | 0.003 (-0.012, 0.017) | 0.730 | **0.019 (0.010, 0.029)** | **< 0.001 ***** |
| Q4 | **0.022 (0.006, 0.037)** | **0.007 **** | **0.024 (0.014, 0.034)** | **< 0.001 ***** |
| Q5 (highest) | **0.070 (0.052, 0.089)** | **< 0.001 ***** | **0.036 (0.026, 0.046)** | **< 0.001 ***** |
| **Education** | | | | |
| Primary education | **Reference** | | | |
| Secondary education | **0.095 (0.074, 0.116)** | **< 0.001 ***** | **0.056 (0.041, 0.070)** | **< 0.001 ***** |
| Higher education | **0.076 (0.056, 0.097)** | **< 0.001 ***** | **0.019 (0.005, 0.033)** | **0.009 **** |
| **ACE** | | | | |
| Number of ACEs | **-0.095 (-0.115, -0.074)** | **< 0.001 ***** | **-0.124 (-0.139, -0.109)** | **< 0.001 ***** |
| **Smoke** | | | | |
| None | **Reference** | | | |
| Former or current | **-0.038 (-0.061, -0.014)** | **0.002 **** | **-0.030 (-0.048, -0.012)** | **0.001 **** |
| **Drink** | | | | |
| None | **Reference** | | | |
| Former or current | **0.018 (0.003, 0.033)** | **0.017 *** | 0.005 (-0.006, 0.016) | 0.355 |
| **Age** | | | | |
| Age | **-0.197 (-0.220, -0.174)** | **< 0.001 ***** | **-0.221 (-0.238, -0.205)** | **< 0.001 ***** |
| **Gender** | | | | |
| Male | **Reference** | | | |
| Female | **-0.199 (-0.226, -0.172)** | **< 0.001 ***** | **-0.224 (-0.244, -0.204)** | **< 0.001 ***** |
| **Marital status** | | | | |
| Unmarried | **Reference** | | | |
| Married | 0.009 (-0.008, 0.026) | 0.293 | **0.027 (0.014, 0.039)** | **< 0.001 ***** |
| **Regional distribution** | | | | |
| West | **Reference** | | | |
| Northeast | 0.015 (-0.007, 0.036) | 0.193 | 0.013 (-0.002, 0.028) | 0.084 |
| Central | **-0.034 (-0.057, -0.010)** | **0.005 **** | 0.010 (-0.007, 0.026) | 0.245 |
| East | **0.044 (0.020, 0.068)** | **< 0.001 ***** | **0.078 (0.062, 0.095)** | **< 0.001 ***** |
| **Random Effects** | | | | |
|  | Variance (SD) | Correlation | Variance (SD) | Correlation |
| **ID (Intercept)** | 36.37 (6.03) |  | 35.37 (5.95) |  |
| **Wave (Slope)** | 0.29 (0.53) | -0.30 | 0.32 (0.56) | -0.35 |
| **Model fits** | | | | |
| **R^2^** | **Marginal** | **Conditional** | **Marginal** | **Conditional** |
|  | 0.25 | 0.63 | 0.26 | 0.61 |

Note: β, standardized coefficient; CI: confidence interval; SD, standard deviation; AFE: Age-Friendly Environments; ACEs: adverse childhood experience.

Marginal: fixed effects; Conditional: fixed and random effects.

Adjusted for chronic diseases, teeth, household income, education, ACE, smoke, drink, age, gender, marital status, and geographic distribution.

* p < .05, ** p < .01, *** p < .001.

Table S8. Sensitivity analysis of associations between AFE and Healthy Ageing stratified by urban and rural in CLPM and RI-CLPM

| **Urban-rural disparities** | **Urban** | | **Rural** | |
| --- | --- | --- | --- | --- |
|  | **Effect size (95% CI)** | ***p*** | **Effect size (95% CI)** | ***p*** |
| **CLPM** | | | | |
| **Unstandardized model results** | | | | |
| AFE→HA | **0.791 (0.692, 0.889)** | **< 0.001 ***** | **0.702 (0.626, 0.777)** | **< 0.001 ***** |
| HA→AFE | **0.019 (0.018, 0.021)** | **< 0.001 ***** | **0.017 (0.016, 0.018)** | **< 0.001 ***** |
| **Standardized model results** | | | | |
| AFE1→HA2 | **0.087 (0.076, 0.098)** | **< 0.001 ***** | **0.072 (0.064, 0.079)** | **< 0.001 ***** |
| AFE2→HA3 | **0.092 (0.081, 0.104)** | **< 0.001 ***** | **0.078 (0.069, 0.086)** | **< 0.001 ***** |
| AFE3→HA4 | **0.098 (0.086, 0.110)** | **< 0.001 ***** | **0.083 (0.074, 0.092)** | **< 0.001 ***** |
| HA1→AFE2 | **0.163 (0.148, 0.178)** | **< 0.001 ***** | **0.153 (0.141, 0.164)** | **< 0.001 ***** |
| HA2→AFE3 | **0.166 (0.150, 0.181)** | **< 0.001 ***** | **0.155 (0.144, 0.166)** | **< 0.001 ***** |
| HA3→AFE4 | **0.156 (0.142, 0.171)** | **< 0.001 ***** | **0.140 (0.130, 0.151)** | **< 0.001 ***** |
| **RI-CLPM** | | | | |
| **Unstandardized model results** | | | | |
| AFE→HA | **0.172 (0.036, 0.309)** | **0.038 *** | 0.120 (0.017, 0.223) | 0.055 |
| HA→AFE | 0.000 (-0.004, 0.003) | 0.815 | 0.001 (-0.002, 0.003) | 0.616 |
| **Standardized model results** | | | | |
| AFE1→HA2 | **0.026 (0.005, 0.047)** | **0.038 *** | 0.017 (0.002, 0.031) | 0.055 |
| AFE2→HA3 | **0.028 (0.006, 0.051)** | **0.038 *** | 0.019 (0.003, 0.035) | 0.056 |
| AFE3→HA4 | **0.028 (0.006, 0.051)** | **0.038 *** | 0.020 (0.003, 0.037) | 0.056 |
| HA1→AFE2 | -0.003 (-0.027, 0.021) | 0.815 | 0.005 (-0.012, 0.022) | 0.616 |
| HA2→AFE3 | -0.003 (-0.024, 0.018) | 0.815 | 0.005 (-0.011, 0.020) | 0.616 |
| HA3→AFE4 | -0.003 (-0.021, 0.016) | 0.815 | 0.004 (-0.009, 0.017) | 0.616 |

Note: CLPM: cross-lagged panel model; RI-CLPM: random-intercept cross-lagged panel model; CI: confidence interval; AFE1, AFE2, AFE3, AFE4: Age-Friendly Environments at Wave 1, 2, 3, 4; HA1, HA2, HA3, HA4: healthy aging at Wave 1, 2, 3, 4.

Adjusted for ACE, education, gender, and regional distribution.

* p < .05, ** p < .01, *** p < .001.

Table S9. Sensitivity analysis of associations between AFE and Healthy Ageing stratified by region in LMM

| **Regional disparities** | **West** | | **Northeast** | | **Central** | | **East** | |
| --- | --- | --- | --- | --- | --- | --- | --- | --- |
|  | **Effect size (95% CI)** | ***p*** | **Effect size (95% CI)** | ***p*** | **Effect size (95% CI)** | ***p*** | **Effect size (95% CI)** | ***p*** |
| **Fixed Effects** | | | | | | | | |
| **AFE** | | | | | | | | |
| Number of AFEs | **0.079 (0.066, 0.092)** | **< 0.001 ***** | **0.038 (0.010, 0.066)** | **0.007 **** | **0.091 (0.078, 0.105)** | **< 0.001 ***** | **0.101 (0.088, 0.114)** | **< 0.001 ***** |
| **Chronic diseases** | | | | | | | | |
| Number of chronic diseases | **-0.231 (-0.248, -0.213)** | **< 0.001 ***** | **-0.325 (-0.363, -0.287)** | **< 0.001 ***** | **-0.229 (-0.247, -0.210)** | **< 0.001 ***** | **-0.226 (-0.243, -0.208)** | **< 0.001 ***** |
| **Teeth** | | | | | | | | |
| Tooth loss | **Reference** | | | | | | | |
| No tooth loss | **0.040 (0.024, 0.057)** | **< 0.001 ***** | 0.014 (-0.020, 0.048) | 0.417 | **0.041 (0.023, 0.058)** | **< 0.001 ***** | **0.041 (0.024, 0.058)** | **< 0.001 ***** |
| **Household income** | | | | | | | |  |
| Q1 (lowest) | **Reference** | | | | | | | |
| Q2 | -0.002 (-0.016, 0.012) | 0.803 | -0.011 (-0.043, 0.020) | 0.478 | 0.009 (-0.005, 0.024) | 0.204 | 0.000 (-0.014, 0.014) | 0.968 |
| Q3 | **0.022 (0.009, 0.036)** | **0.002 **** | 0.004 (-0.027, 0.036) | 0.778 | 0.007 (-0.008, 0.022) | 0.346 | 0.013 (-0.001, 0.028) | 0.069 |
| Q4 | **0.034 (0.019, 0.048)** | **< 0.001 ***** | 0.008 (-0.025, 0.040) | 0.648 | **0.020 (0.005, 0.035)** | **0.008 **** | **0.017 (0.002, 0.032)** | **0.026 *** |
| Q5 (highest) | **0.047 (0.031, 0.062)** | **< 0.001 ***** | **0.073 (0.035, 0.111)** | **< 0.001 ***** | **0.049 (0.032, 0.065)** | **< 0.001 ***** | **0.056 (0.039, 0.073)** | **< 0.001 ***** |
| **Education** | | | | | | | | |
| Primary education | **Reference** | | | | | | | |
| Secondary education | **0.080 (0.059, 0.101)** | **< 0.001 ***** | **0.083 (0.037, 0.130)** | **< 0.001 ***** | **0.073 (0.051, 0.096)** | **< 0.001 ***** | **0.059 (0.037, 0.080)** | **< 0.001 ***** |
| Higher education | **0.049 (0.029, 0.069)** | **< 0.001 ***** | 0.038 (-0.006, 0.083) | 0.089 | **0.066 (0.043, 0.088)** | **< 0.001 ***** | **0.038 (0.017, 0.059)** | **< 0.001 ***** |
| **ACE** | | | | | | | | |
| Number of ACEs | **-0.118 (-0.138, -0.097)** | **< 0.001 ***** | **-0.117 (-0.162, -0.073)** | **< 0.001 ***** | **-0.122 (-0.144, -0.100)** | **< 0.001 ***** | **-0.100 (-0.121, -0.079)** | **< 0.001 ***** |
| **Smoke** | | | | | | | | |
| None | **Reference** | | | | | | | |
| Former or current | -0.018 (-0.043, 0.007) | 0.162 | **-0.021 (-0.066, 0.025)** | **0.373** | **-0.070 (-0.098, -0.042)** | **< 0.001 ***** | **-0.027 (-0.053, -0.002)** | **0.037 *** |
| **Drink** | | | | | | | | |
| None | **Reference** | | | | | | | |
| Former or current | -0.006 (-0.021, 0.010) | 0.479 | **0.050 (0.015, 0.085)** | **0.005 **** | -0.003 (-0.019, 0.013) | 0.697 | **0.032 (0.015, 0.048)** | **< 0.001 ***** |
| **Age** | | | | | | | | |
| Age | **-0.191 (-0.214, -0.168)** | **< 0.001 ***** | **-0.250 (-0.299, -0.200)** | **< 0.001 ***** | **-0.225 (-0.249, -0.200)** | **< 0.001 ***** | **-0.215 (-0.238, -0.191)** | **< 0.001 ***** |
| **Gender** | | | | | | | | |
| Male | **Reference** | | | | | | | |
| Female | **-0.210 (-0.238, -0.182)** | **< 0.001 ***** | **-0.112 (-0.164, -0.059)** | **< 0.001 ***** | **-0.248 (-0.280, -0.217)** | **< 0.001 ***** | **-0.215 (-0.244, -0.186)** | **< 0.001 ***** |
| **Marital status** | | | | | | | | |
| Unmarried | **Reference** | | | | | | | |
| Married | **0.028 (0.011, 0.046)** | **0.002 **** | -0.002 (-0.037, 0.033) | 0.927 | 0.003 (-0.016, 0.022) | 0.752 | **0.035 (0.017, 0.054)** | **< 0.001 ***** |
| **Urban-rural distribution** | | | | | | | | |
| Urban | **Reference** | | | | | | | |
| Rural | **-0.108 (-0.129, -0.087)** | **< 0.001 ***** | **-0.111 (-0.159, -0.063)** | **< 0.001 ***** | **-0.063 (-0.085, -0.040)** | **< 0.001 ***** | **-0.078 (-0.100, -0.057)** | **< 0.001 ***** |
| **Random Effects** | | | | | | | | |
|  | Variance (SD) | Correlation | Variance (SD) | Correlation | Variance (SD) | Correlation | Variance (SD) | Correlation |
| **ID (Intercept)** | 35.43 (5.95) |  | 37.38 (6.11) |  | 35.34 (5.94) |  | 35.73 (5.98) |  |
| **Wave (Slope)** | 0.25 (0.50) | -0.33 | 0.35 (0.59) | -0.35 | 0.29 (0.54) | -0.32 | 0.37 (0.61) | -0.34 |
| **Model fits** | | | | | | | | |
| **R^2^** | **Marginal** | **Conditional** | **Marginal** | **Conditional** | **Marginal** | **Conditional** | **Marginal** | **Conditional** |
|  | 0.25 | 0.60 | 0.30 | 0.64 | 0.26 | 0.63 | 0.26 | 0.63 |

Note: β, standardized coefficient; CI: confidence interval; SD, standard deviation; AFE: Age-Friendly Environments; ACEs: adverse childhood experience.

Marginal: fixed effects; Conditional: fixed and random effects.

Adjusted for chronic diseases, teeth, household income, education, ACE, smoke, drink, age, gender, marital status, and urban-rural distribution.

* p < .05, ** p < .01, *** p < .001.

Table S10. Sensitivity analysis of associations between AFE and Healthy Ageing stratified by region in CLPM and RI-CLPM

| **Regional disparities** | **West** | | **Northeast** | | **Central** | | **East** | |
| --- | --- | --- | --- | --- | --- | --- | --- | --- |
|  | **β (95% CI)** | ***p*** | **β (95% CI)** | ***p*** | **β (95% CI)** | ***p*** | **β (95% CI)** | ***p*** |
| **CLPM** | | | | | | | | |
| **Unstandardized model results** | | | | | | | | |
| AFE→HA | **0.600 (0.497, 0.704)** | **< 0.001 ***** | **0.612 (0.390, 0.833)** | **< 0.001 ***** | **0.781 (0.665, 0.897)** | **< 0.001 ***** | **0.836 (0.729, 0.943)** | **< 0.001 ***** |
| HA→AFE | **0.017 (0.015, 0.019)** | **< 0.001 ***** | **0.017 (0.013, 0.021)** | **< 0.001 ***** | **0.020 (0.018, 0.022)** | **< 0.001 ***** | **0.016 (0.014, 0.018)** | **< 0.001 ***** |
| **Standardized model results** | | | | | | | | |
| AFE1→HA2 | **0.065 (0.053, 0.076)** | **< 0.001 ***** | **0.064 (0.041, 0.088)** | **< 0.001 ***** | **0.079 (0.067, 0.091)** | **< 0.001 ***** | **0.087 (0.076, 0.099)** | **< 0.001 ***** |
| AFE2→HA3 | **0.068 (0.056, 0.079)** | **< 0.001 ***** | **0.070 (0.045, 0.095)** | **< 0.001 ***** | **0.085 (0.072, 0.098)** | **< 0.001 ***** | **0.096 (0.084, 0.109)** | **< 0.001 ***** |
| AFE3→HA4 | **0.074 (0.061, 0.087)** | **< 0.001 ***** | **0.072 (0.046, 0.098)** | **< 0.001 ***** | **0.090 (0.077, 0.104)** | **< 0.001 ***** | **0.101 (0.088, 0.114)** | **< 0.001 ***** |
| HA1→AFE2 | **0.154 (0.138, 0.170)** | **< 0.001 ***** | **0.148 (0.115, 0.181)** | **< 0.001 ***** | **0.175 (0.158, 0.192)** | **< 0.001 ***** | **0.141 (0.125, 0.157)** | **< 0.001 ***** |
| HA2→AFE3 | **0.150 (0.135, 0.166)** | **< 0.001 ***** | **0.155 (0.121, 0.190)** | **< 0.001 ***** | **0.184 (0.166, 0.201)** | **< 0.001 ***** | **0.138 (0.122, 0.154)** | **< 0.001 ***** |
| HA3→AFE4 | **0.143 (0.128, 0.158)** | **< 0.001 ***** | **0.143 (0.111, 0.176)** | **< 0.001 ***** | **0.163 (0.147, 0.179)** | **< 0.001 ***** | **0.129 (0.114, 0.144)** | **< 0.001 ***** |
| **RI-CLPM** | | | | | | | | |
| **Unstandardized model results** | | | | | | | | |
| AFE→HA | -0.026 (-0.162, 0.110) | 0.756 | 0.221 (-0.097, 0.540) | 0.253 | 0.172 (0.010, 0.335) | 0.082 | **0.276 (0.125, 0.427)** | **0.003 **** |
| HA→AFE | -0.002 (-0.005, 0.001) | 0.381 | 0.007 (0.000, 0.015) | 0.093 | 0.003 (0.000, 0.007) | 0.121 | -0.001 (-0.005, 0.002) | 0.588 |
| **Standardized model results** | | | | | | | | |
| AFE1→HA2 | -0.004 (-0.024, 0.017) | 0.756 | 0.033 (-0.014, 0.080) | 0.254 | 0.023 (0.001, 0.044) | 0.080 | **0.040 (0.018, 0.061)** | **0.003 **** |
| AFE2→HA3 | -0.004 (-0.026, 0.018) | 0.756 | 0.037 (-0.016, 0.091) | 0.253 | 0.026 (0.001, 0.050) | 0.082 | **0.046 (0.021, 0.071)** | **0.003 **** |
| AFE3→HA4 | -0.004 (-0.028, 0.019) | 0.756 | 0.034 (-0.015, 0.084) | 0.254 | 0.027 (0.001, 0.052) | 0.082 | **0.045 (0.020, 0.069)** | **0.003 **** |
| HA1→AFE2 | -0.013 (-0.037, 0.011) | 0.381 | 0.055 (0.001, 0.109) | 0.094 | 0.025 (-0.002, 0.052) | 0.121 | -0.008 (-0.033, 0.017) | 0.588 |
| HA2→AFE3 | -0.011 (-0.031, 0.009) | 0.381 | 0.049 (0.001, 0.097) | 0.092 | 0.025 (-0.002, 0.052) | 0.123 | -0.007 (-0.030, 0.015) | 0.588 |
| HA3→AFE4 | -0.010 (-0.028, 0.009) | 0.381 | 0.042 (0.001, 0.083) | 0.094 | 0.020 (-0.001, 0.041) | 0.123 | -0.006 (-0.026, 0.013) | 0.588 |

Note: CLPM: cross-lagged panel model; RI-CLPM: random-intercept cross-lagged panel model; CI: confidence interval; AFE1, AFE2, AFE3, AFE4: Age-Friendly Environments at Wave 1, 2, 3, 4; HA1, HA2, HA3, HA4: healthy aging at Wave 1, 2, 3, 4.

Adjusted for ACE, education, gender, and urban-rural distribution.

* p < .05, ** p < .01, *** p < .001.

Table S11. Sensitivity analysis of associations between AFE and Health Ageing scores derived from summation method in LMM

| **Health Ageing scores derived from summation method** | **β (95% CI)** | ***p*** |
| --- | --- | --- |
| **Fixed Effects** | | |
| **AFE** | | |
| Number of AFEs | **0.090 (0.083, 0.097)** | **< 0.001 ***** |
| **Chronic diseases** | | |
| Number of chronic diseases | **-0.221 (-0.230, -0.211)** | **< 0.001 ***** |
| **Teeth** | | |
| Tooth loss | Reference | |
| No tooth loss | **0.045 (0.035, 0.054)** | **< 0.001 ***** |
| **Household income** | | |
| Q1 (lowest) | Reference | |
| Q2 | 0.002 (-0.006, 0.009) | 0.629 |
| Q3 | **0.015 (0.007, 0.022)** | **< 0.001 ***** |
| Q4 | **0.024 (0.016, 0.032)** | **< 0.001 ***** |
| Q5 (highest) | **0.054 (0.045, 0.063)** | **< 0.001 ***** |
| **Education** | | |
| Primary education | Reference | |
| Secondary education | **0.068 (0.056, 0.079)** | **< 0.001 ***** |
| Higher education | **0.042 (0.031, 0.053)** | **< 0.001 ***** |
| **ACE** | | |
| Number of ACEs | **-0.109 (-0.121, -0.098)** | **< 0.001 ***** |
| **Smoke** | | |
| None | Reference | |
| Former or current | -0.013 (-0.026, 0.001) | 0.071 |
| **Drink** | | |
| None | Reference | |
| Former or current | **0.010 (0.002, 0.019)** | **0.015 *** |
| **Age** | | |
| Age | **-0.203 (-0.215, -0.190)** | **< 0.001 ***** |
| **Gender** | | |
| Male | Reference | |
| Female | **-0.172 (-0.188, -0.157)** | **< 0.001 ***** |
| **Marital status** | | |
| Unmarried | Reference | |
| Married | **0.034 (0.024, 0.044)** | **< 0.001 ***** |
| **Urban-rural distribution** | | |
| Urban | Reference | |
| Rural | **-0.092 (-0.103, -0.080)** | **< 0.001 ***** |
| **Regional distribution** | | |
| West | Reference | |
| Northeast | **0.022 (0.010, 0.034)** | **< 0.001 ***** |
| Central | 0.001 (-0.012, 0.014) | 0.909 |
| East | **0.068 (0.055, 0.081)** | **< 0.001 ***** |
| **Random Effects** | | |
|  | Variance (SD) | Correlation |
| **ID (Intercept)** | 6.00 (2.45) |  |
| **Wave (Slope)** | 0.10 (0.32) | 0.03 |
| **Model fits** | | |
| **R^2^** | **Marginal** | **Conditional** |
|  | 0.29 | 0.67 |

Note: β, standardized coefficient; CI: confidence interval; SD, standard deviation; AFE: Age-Friendly Environments; ACEs: adverse childhood experience.

Marginal: fixed effects; Conditional: fixed and random effects.

Adjusted for chronic diseases, teeth, household income, education, ACE, smoke, drink, age, gender, marital status, urban-rural distribution, and geographic distribution.

* p < .05, ** p < .01, *** p < .001.

Table S12.Sensitivity analysis of associations between AFE and Health Ageing scores derived from summation method in CLPM and RI-CLPM

| **Health Ageing scores derived from summation method** | **Effect size (95% CI)** | ***p*** |  |
| --- | --- | --- | --- |
|  |  |  |  |
| **CLPM** | | |  |
| **Unstandardized model results** | | |  |
| AFE→HA | **0.322 (0.294, 0.350)** | **< 0.001 ***** |  |
| HA→AFE | **0.043 (0.040, 0.045)** | **< 0.001 ***** |  |
| **Standardized model results** | | |  |
| AFE1→HA2 | **0.078 (0.071, 0.084)** | **< 0.001 ***** |  |
| AFE2→HA3 | **0.077 (0.070, 0.083)** | **< 0.001 ***** |  |
| AFE3→HA4 | **0.076 (0.069, 0.082)** | **< 0.001 ***** |  |
| HA1→AFE2 | **0.159 (0.150, 0.167)** | **< 0.001 ***** |  |
| HA2→AFE3 | **0.164 (0.155, 0.173)** | **< 0.001 ***** |  |
| HA3→AFE4 | **0.164 (0.155, 0.173)** | **< 0.001 ***** |  |
| **RI-CLPM** | | |  |
| **Unstandardized model results** | | |  |
| AFE→HA | **0.082 (0.042, 0.122)** | **0.001 **** |  |
| HA→AFE | 0.002 (-0.002, 0.007) | 0.425 |  |
| **Standardized model results** | | |  |
| AFE1→HA2 | **0.028 (0.014, 0.041)** | **0.001 **** |  |
| AFE2→HA3 | **0.027 (0.014, 0.040)** | **0.001 **** |  |
| AFE3→HA4 | **0.025 (0.013, 0.037)** | **0.001 **** |  |
| HA1→AFE2 | 0.007 (-0.007, 0.021) | 0.424 |  |
| HA2→AFE3 | 0.006 (-0.007, 0.019) | 0.425 |  |
| HA3→AFE4 | 0.006 (-0.007, 0.019) | 0.425 |  |

Note: CLPM: cross-lagged panel model; RI-CLPM: random-intercept cross-lagged panel model; CI: confidence interval; AFE1, AFE2, AFE3, AFE4: Age-Friendly Environments at Wave 1, 2, 3, 4; HA1, HA2, HA3, HA4: healthy aging at Wave 1, 2, 3, 4.

Adjusted for ACE, education, gender, urban-rural distribution, and regional distribution.

* p < .05, ** p < .01, *** p < .001.

**References**

1. World Health Organization. Measuring the age-friendliness of cities: a guide to using core indicators. 2023/5/12. <https://www.who.int/china/publications-detail/9789241509695>. Accessed 12 May 2023.

2. Lai M-M, Lein S-Y, Lau S-H, Lai M-L. Modeling Age-Friendly Environment, Active Aging, and Social Connectedness in an Emerging Asian Economy. J Aging Res. 2016;2016:2052380. doi:10.1155/2016/2052380.

3. Park S, Lee S. Age-friendly environments and life satisfaction among South Korean elders: person-environment fit perspective. Aging Ment Health. 2017;21:693–702. doi:10.1080/13607863.2016.1154011.

4. Nieboer AP, Cramm JM. Age-Friendly Communities Matter for Older People’s Well-Being. J Happiness Stud. 2018;19:2405–20. doi:10.1007/s10902-017-9923-5.

5. Paiva NM de, Daniel F, Da Silva AG, Vicente HT. Age-friendly Coimbra city, Portugal, perception and quality of life in a sample of elderly persons. [Age-friendly Coimbra city, Portugal, perception and quality of life in a sample of elderly persons]. Cien Saude Colet. 2019;24:1473–82. doi:10.1590/1413-81232018244.08902017.

6. Yu J, Ma G, Cai S. Disparities in the provision of aging-friendly communities in old and new urban neighborhoods in China. ECAM. 2019;26:1277–93. doi:10.1108/ECAM-03-2018-0092.

7. Yu R, Wong M, Woo J. Perceptions of Neighborhood Environment, Sense of Community, and Self-Rated Health: an Age-Friendly City Project in Hong Kong. J Urban Health. 2019;96:276–88. doi:10.1007/s11524-018-00331-3.

8. Garner IW, Holland CA. Age-friendliness of living environments from the older person’s viewpoint: development of the Age-Friendly Environment Assessment Tool. Age Ageing. 2020;49:193–8. doi:10.1093/ageing/afz146.

9. Ward M, Gibney S, O’Callaghan D, Shannon S. Age-Friendly Environments, Active Lives? Associations Between the Local Physical and Social Environment and Physical Activity Among Adults Aged 55 and Older in Ireland. J Aging Phys Act. 2020;28:140–8. doi:10.1123/japa.2019-0012.

10. Lei P, Feng Z. Age-friendly neighbourhoods and depression among older people in China: Evidence from China Family Panel Studies. J Affect Disord. 2021;286:187–96. doi:10.1016/j.jad.2021.02.081.

11. Park S-Y, Kim M, Chung S. Age-friendly environments and depressive symptoms among Korean adults: The mediating effects of loneliness. Aging Ment Health. 2021;25:1060–70. doi:10.1080/13607863.2020.1755827.

12. Tang JYM, Chui CHK, Lou VWQ, Chiu RLH, Kwok R, Tse M, et al. The Contribution of Sense of Community to the Association Between Age-Friendly Built Environment and Health in a High-Density City: A Cross-Sectional Study of Middle-Aged and Older Adults in Hong Kong. J Appl Gerontol. 2021;40:1687–96. doi:10.1177/0733464821991298.

13. Jagroep W, Cramm JM, Denktaș S, Nieboer AP. Age-friendly neighbourhoods and physical activity of older Surinamese individuals in Rotterdam, the Netherlands. PLoS One. 2022;17:e0261998. doi:10.1371/journal.pone.0261998.

14. John DH, Gunter K. engAGE in Community: Using Mixed Methods to Mobilize Older People to Elucidate the Age-Friendly Attributes of Urban and Rural Places. J Appl Gerontol. 2016;35:1095–120. doi:10.1177/0733464814566679.

15. Wang Y, Gonzales E, Morrow-Howell N. Applying WHO’s Age-Friendly Communities Framework to a National Survey in China. J Gerontol Soc Work. 2017;60:215–31. doi:10.1080/01634372.2017.1292980.

16. Wang Y, Chen Y-C, Shen H-W, Morrow-Howell N. Neighborhood and Depressive Symptoms: A Comparison of Rural and Urban Chinese Older Adults. Gerontologist. 2018;58:68–78. doi:10.1093/geront/gnx063.

17. Park S, Lee S. Heterogeneous Age-Friendly Environments among Age-Cohort Groups. Sustainability. 2018;10:1269. doi:10.3390/su10041269.

18. Zuluaga MC, Guallar-Castillón P, Conthe P, Rodríguez-Pascual C, Graciani A, León-Muñoz LM, et al. Housing conditions and mortality in older patients hospitalized for heart failure. Am Heart J. 2011;161:950–5. doi:10.1016/j.ahj.2011.03.002.

19. Choi YJ. Age-Friendly Features in Home and Community and the Self-Reported Health and Functional Limitation of Older Adults: the Role of Supportive Environments. J Urban Health. 2020;97:471–85. doi:10.1007/s11524-020-00462-6.

20. Wong M, Chau PH, Cheung F, Phillips DR, Woo J. Comparing the Age-Friendliness of Different Neighbourhoods Using District Surveys: An Example from Hong Kong. PLoS One. 2015;10:e0131526. doi:10.1371/journal.pone.0131526.

21. Kim K, Buckley T, Burnette D, Kim S, Cho S. Measurement Indicators of Age-Friendly Communities: Findings From the AARP Age-Friendly Community Survey. Gerontologist. 2022;62:e17-e27. doi:10.1093/geront/gnab055.

22. Au A, Lai DWL, Yip H-M, Chan S, Lai S, Chaudhury H, et al. Sense of Community Mediating Between Age-Friendly Characteristics and Life Satisfaction of Community-Dwelling Older Adults. Front Psychol. 2020;11:86. doi:10.3389/fpsyg.2020.00086.

23. Samei Sis S, Safaeeian A, Azizi Zeinalhajlou A, Matlabi H. Viewpoints of Older People Toward the Features of Age-Friendly Communities: Map for Charting Progress in Tabriz, Iran. Journal of Aging and Environment. 2022;36:73–89. doi:10.1080/26892618.2020.1859037.

24. Aung MN, Koyanagi Y, Ueno S, Tiraphat S, Yuasa M. A Contemporary Insight into an Age-Friendly Environment Contributing to the Social Network, Active Ageing and Quality of Life of Community Resident Seniors in Japan. Journal of Aging and Environment. 2021;35:145–60. doi:10.1080/26892618.2020.1813232.

25. Choi YJ. Understanding Aging in Place: Home and Community Features, Perceived Age-Friendliness of Community, and Intention Toward Aging in Place. Gerontologist. 2022;62:46–55. doi:10.1093/geront/gnab070.

26. Yu J, Ma G, Ding W, Mao J, Wang J. Structural model for the relationships between age-friendly communities and quality of life of older adults in Hefei, China. ECAM. 2021;29:1376–95. doi:10.1108/ECAM-08-2020-0647.

27. Zhang X, Warner ME, Wethington E. Can Age-Friendly Planning Promote Equity in Community Health Across the Rural-Urban Divide in the US? International journal of environmental research and public health 2020. doi:10.3390/ijerph17041275.

28. Sun J, Lyu S, Li C, Coyte PC. The contribution of Urban and Rural Resident Basic Medical Insurance to income-related inequality in depression among middle-aged and older adults: Evidence from China. J Affect Disord. 2021;293:168–75. doi:10.1016/j.jad.2021.06.027.

29. Mo S, Wang Y, Peng M, Wang Q, Zheng H, Zhan Y, et al. Sex disparity in cognitive aging related to later-life exposure to ambient air pollution. Sci Total Environ. 2023;886:163980. doi:10.1016/j.scitotenv.2023.163980.

30. Ministry of Ecology and Environment of the People’s Republic of China. Environmental Air Quality Standards（GB 3095—2012）. 2023/5/12. <https://www.mee.gov.cn/ywgz/fgbz/bz/bzwb/dqhjbh/dqhjzlbz/201203/t20120302_224165.htm>. Accessed 12 May 2023.

31. Zhao Y, Atun R, Oldenburg B, McPake B, Tang S, Mercer SW, et al. Physical multimorbidity, health service use, and catastrophic health expenditure by socioeconomic groups in China: an analysis of population-based panel data. Lancet Glob Health. 2020;8:e840-e849. doi:10.1016/S2214-109X(20)30127-3.

32. Brossoie N, Hwang E, Song K, Jeong JW, Young-Woo K. Assessing Age-Friendliness: Individualistic vs. Collectivistic Cultures. J Aging Soc Policy. 2022;34:311–34. doi:10.1080/08959420.2022.2049569.

33. Hsu H-C. Associations of City-Level Active Aging and Age Friendliness with Well-Being among Older Adults Aged 55 and Over in Taiwan. International journal of environmental research and public health 2020. doi:10.3390/ijerph17124304.

34. Wong M, Yu R, Woo J. Effects of Perceived Neighbourhood Environments on Self-Rated Health among Community-Dwelling Older Chinese. International journal of environmental research and public health 2017. doi:10.3390/ijerph14060614.

35. Del Barrio E, Pinzón S, Marsillas S, Garrido F. Physical Environment vs. Social Environment: What Factors of Age-Friendliness Predict Subjective Well-Being in Men and Women? International journal of environmental research and public health 2021. doi:10.3390/ijerph18020798.

36. Hwang E, Brossoie N, Jeong JW, Song K. The Impacts of the Neighborhood Built Environment on Social Capital for Middle-Aged and Elderly Koreans. Sustainability. 2021;13:756. doi:10.3390/su13020756.

37. Vecchio G, Tiznado-Aitken I, Castillo B, Steiniger S. Fair transport policies for older people: accessibility and affordability of public transport in Santiago, Chile. Transportation (Amst). 2022:1–27. doi:10.1007/s11116-022-10346-0.

38. National Bureau of Statistics. Consumer Price Index. 2023/5/12. <https://data.stats.gov.cn/search.htm?s=%E5%B9%B4%E5%BA%A6cpi>. Accessed 12 May 2023.

39. Hu B. Childhood adversity and healthy ageing: a study of the Chinese older population. EUR J AGEING. 2021;18:523–35. doi:10.1007/s10433-021-00608-8.

40. National Bureau of Statistics. Method of Dividing the Eastern, Western, Central, and Northeastern Regions. 2011. <http://www.stats.gov.cn/zt_18555/zthd/sjtjr/dejtjkfr/tjkp/202302/t20230216_1909741.htm>. Accessed 22 Aug 2023.
